# Engineering SARS-CoV-2 neutralizing antibodies for increased potency and reduced viral escape pathways

**DOI:** 10.1016/j.isci.2022.104914

**Published:** 2022-08-11

**Authors:** Fangzhu Zhao, Celina Keating, Gabriel Ozorowski, Namir Shaabani, Irene M. Francino-Urdaniz, Shawn Barman, Oliver Limbo, Alison Burns, Panpan Zhou, Michael J. Ricciardi, Jordan Woehl, Quoc Tran, Hannah L. Turner, Linghang Peng, Deli Huang, David Nemazee, Raiees Andrabi, Devin Sok, John R. Teijaro, Timothy A. Whitehead, Andrew B. Ward, Dennis R. Burton, Joseph G. Jardine

**Affiliations:** 1 Department of Immunology and Microbiology, The Scripps Research Institute, La Jolla, CA 92037, USA; 2 IAVI Neutralizing Antibody Center, The Scripps Research Institute, La Jolla, CA 92037, USA; 3 Consortium for HIV/AIDS Vaccine Development (CHAVD), The Scripps Research Institute, La Jolla, CA 92037, USA; 4 Department of Chemical and Biological Engineering, University of Colorado, Boulder, CO 80305, USA; 5 Department of Integrative Structural and Computational Biology, The Scripps Research Institute, La Jolla, CA 92037, USA; 6 IAVI, New York, NY 10004, USA; 7 Department of Pathology, George Washington University, Washington, DC 20052, USA; 8 Ragon Institute of Massachusetts General Hospital, Massachusetts Institute of Technology, and Harvard University, Cambridge, MA 02139, USA

**Keywords:** Immunology, Virology, Structural biology

## Abstract

The rapid spread of SARS-CoV-2 variants poses a constant threat of escape from monoclonal antibody and vaccine countermeasures. Mutations in the ACE2 receptor binding site on the surface S protein have been shown to disrupt antibody binding and prevent viral neutralization. Here, we used a directed evolution-based approach to engineer three neutralizing antibodies for enhanced binding to S protein. The engineered antibodies showed increased *in vitro* functional activity in terms of neutralization potency and/or breadth of neutralization against viral variants. Deep mutational scanning revealed that higher binding affinity reduces the total number of viral escape mutations. Studies in the Syrian hamster model showed two examples where the affinity-matured antibody provided superior protection compared to the parental antibody. These data suggest that monoclonal antibodies for antiviral indications would benefit from affinity maturation to reduce viral escape pathways and appropriate affinity maturation in vaccine immunization could help resist viral variation.

## Highlights

• Cryo-EM analysis of Class 2 and Class 3 SARS-CoV-2 neutralizing monoclonal antibodies
• Engineered antibodies with higher affinity neutralize emerging SARS-CoV-2 variants
• The higher affinity antibodies reduce the pathways for viral escape
• Engineered antibodies improve protection in hamster model

## Graphical abstract

**Figure:**
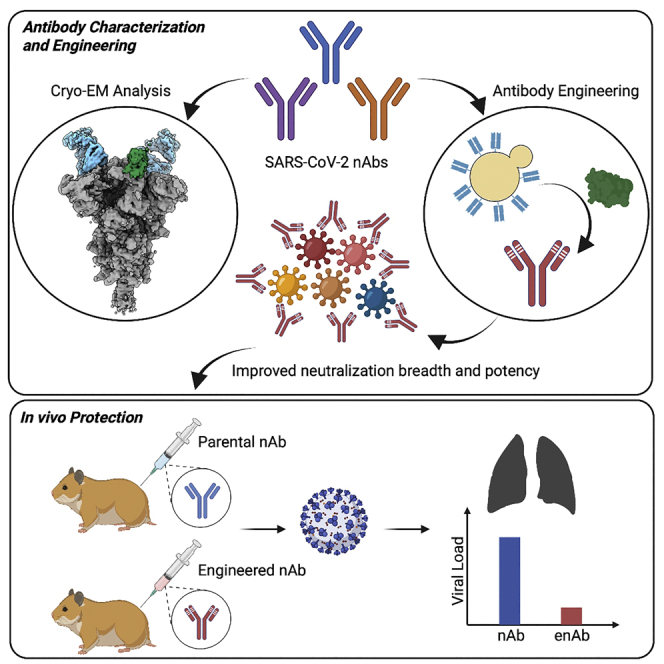

## Introduction

Over the past two years, severe acute respiratory syndrome coronavirus 2 (SARS-CoV-2) has had devastating consequences for global health and economies. Following the discovery of the disease, there was a rush to produce protective vaccines and therapeutics. Multiple highly effective vaccines have been developed that elicit immune responses against the SARS-CoV-2 spike (S) trimer (Creech et al., 2021). The protective mechanisms for the coronavirus disease 2019 (COVID-19) vaccines are still being deduced, however, several analyses have found that the elicitation of neutralizing antibodies (nAbs) correlates with protection (Feng et al., 2021; McMahan et al., 2021), a finding consistent with many other successful antiviral vaccines (Plotkin, 2010). nAbs have been identified that target several distinct epitopes on the S trimer, but the majority of nAbs target the receptor binding domain (RBD) (Barnes et al., 2020; Piccoli et al., 2020). Although vaccines are undisputedly the most effective strategy for control of COVID-19, recombinantly produced nAbs offer the potential to supplement prophylactic coverage in populations that respond poorly to vaccines, e.g., immunocompromised individuals can be administered a post-exposure prophylactic and it can be used therapeutically to prevent hospitalization (Copin et al., 2021; Weinreich et al., 2021).

One of the unique challenges in using a neutralizing monoclonal antibody (mAb) for antiviral indications is addressing existing viral diversity and the high mutational propensity in viruses that can give rise to resistant viral variants. Since the discovery of SARS-CoV-2 in 2019, thousands of viral variants containing synonymous and non-synonymous mutations have been documented (https://nextstrain.org/ncov/gisaid/global). A growing number of these new variants (termed “Variants of Interest” or VOIs, and “Variants of Concern” or VOCs) contain mutations that increase infectivity and/or allow viral escape from monoclonal nAbs elicited against the original SARS-CoV-2 (Wang et al., 2021a,2021b; Wibmer et al., 2021; Yuan et al., 2021a). Several strategies are commonly used to mitigate the likelihood of viral escape from nAbs. Investigators often select antibodies that target functionally important and conserved regions, reducing the number of mutations that can allow viral escape without incurring a fitness cost (Fedry et al., 2021; Li et al., 2021; Liu et al., 2020). It is also common to use cocktails of at least two nAbs targeting different epitopes, so multiple mutations are necessary for viral escape (Julg et al., 2017; Starr et al., 2021a,2021b; Xu et al., 2017). A third approach that is less well explored is to *in vitro* affinity mature the nAb against the target antigen to increase the binding affinity, helping to mitigate the impact of the viral mutations (Rappazzo et al., 2021). Here, we explore how increased binding affinity impacts the *in vitro* neutralization breadth and potency of three COVID-19 nAbs, CC12.1, CC6.30 and CC6.33 (Rogers et al., 2020), and how these affinity improvements impact the *in vivo* protective capability of these nAbs.

## Results

### Structural analysis of nAbs CC6.30 and CC6.33

**Figure 1:**
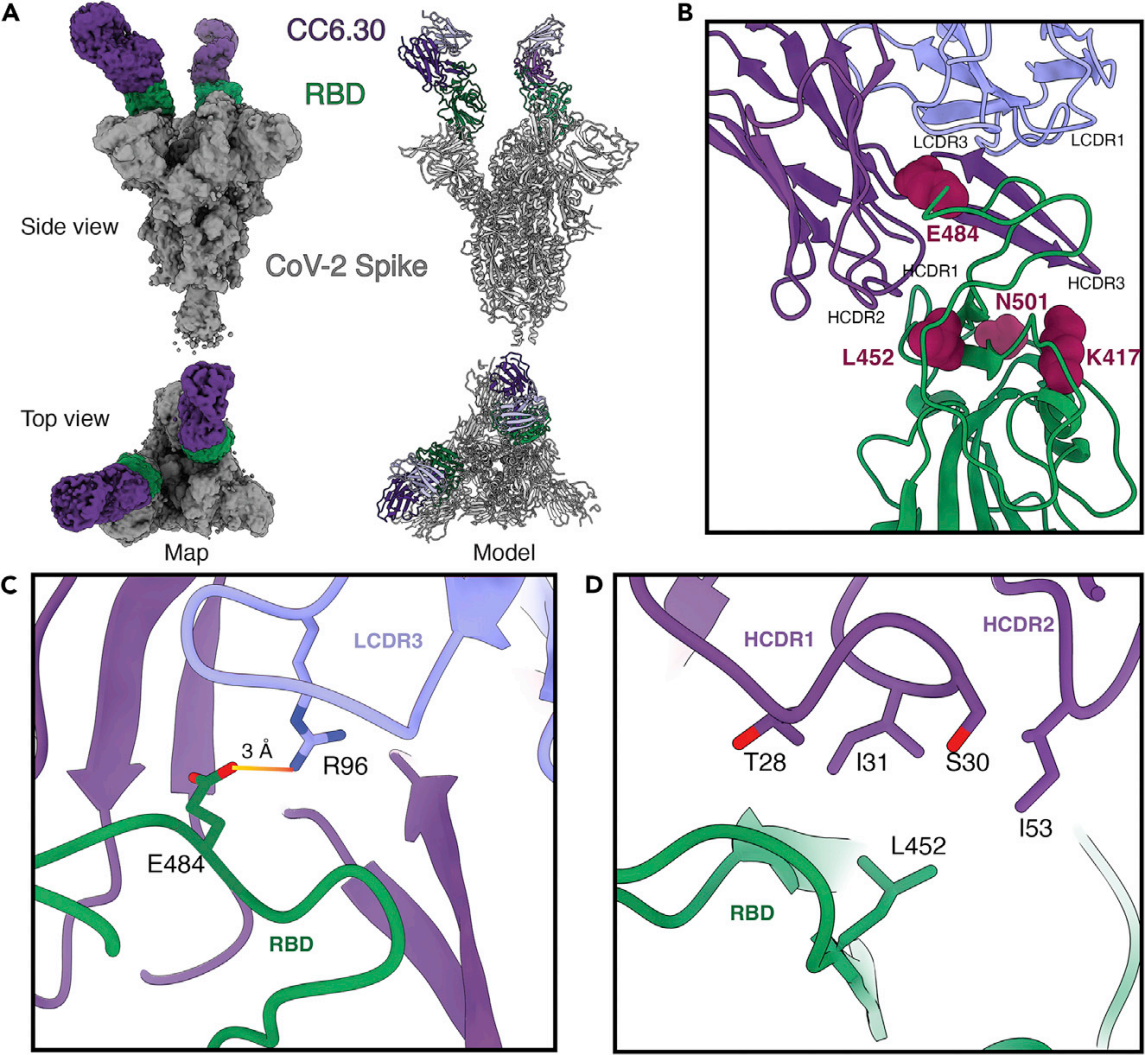
Structural characterization of nAb CC6.30 (A) Cryo-EM map and model, with CC6.30 Fab and Spike RBD colored for clarity. (B) Overview of CC6.30/RBD interface with key antibody CDRs and SARS-CoV-2 mutation sites labeled. (C) Saltbridge between LCDR3 R96 and RBD E484. (D) Hydrophobic interactions between RBD L452 and side chains of HCDR1 and HCDR2. See also Figure S1 and Table S1.

**Figure 2:**
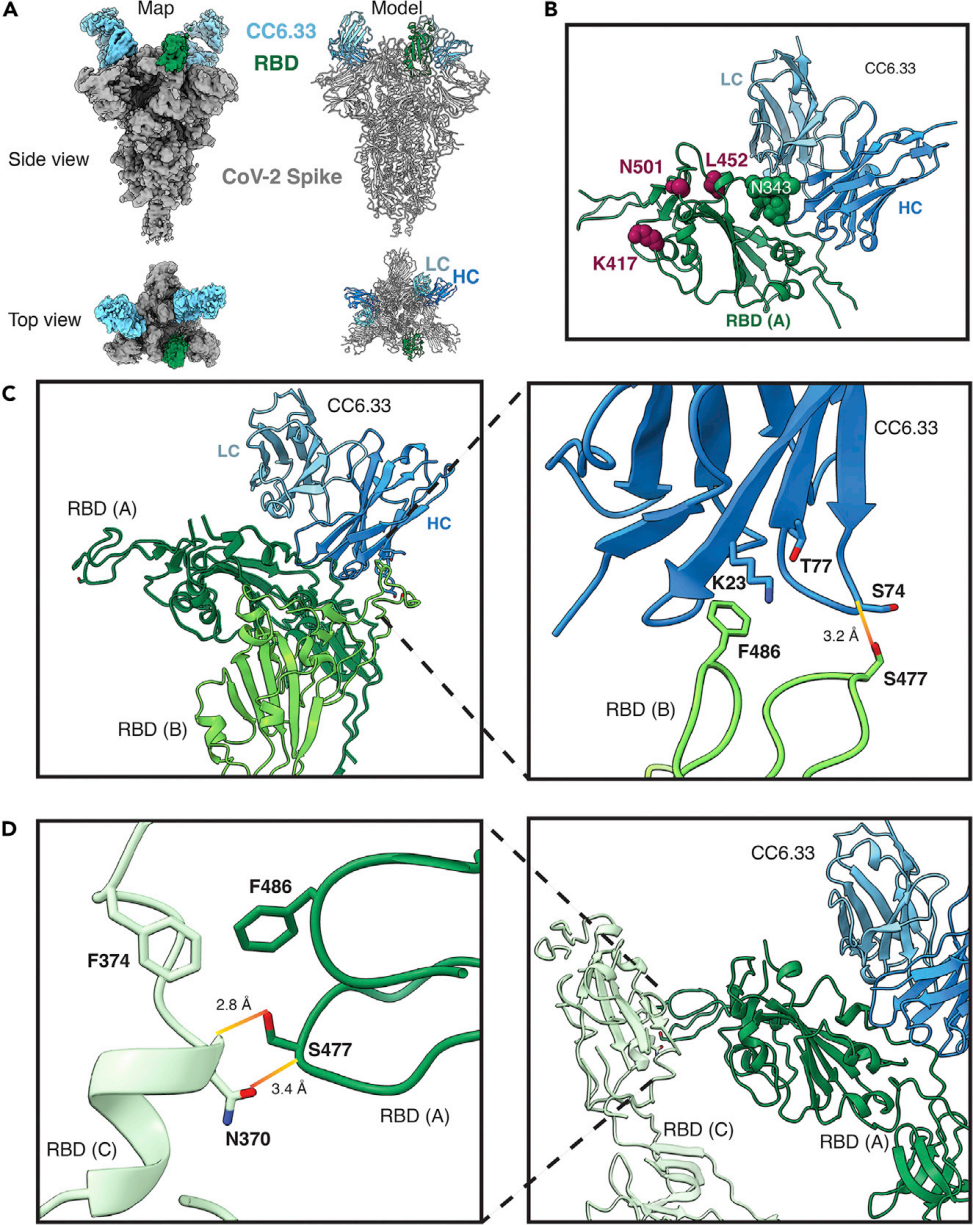
Structural characterization of nAb CC6.33 (A) Cryo-EM map and model, with CC6.33 Fab and Spike RBD colored for clarity. (B) Overview of CC6.33/RBD interface that is centered on the Spike N343 glycan. Common mutation sites in SARS-CoV-2 variants are highlighted and are distal from the CC6.33 epitope. The RBD ridge of a neighboring protomer interacts with framework regions of CC6.33 (C), in a manner similar to the ridge of a down-RBD supporting a neighboring up-RBD (D). See also Figure S2 and Table S2.

Previously, we reported the structure of nAb CC12.1, which binds to the RBS-A or Class 1 epitope site and competes directly with angiotensin-converting enzyme 2 (ACE2) (Barnes et al., 2020; Yuan et al., 2020,2021b) but specificities of two other nAbs of interest, CC6.30 and CC6.33, were limited to epitope-binning data (Rogers et al., 2020). To better understand the molecular contacts of antibodies, we used cryoelectron microscopy (cryoEM) to solve the structures of two of the antibodies in complex with stabilized SARS-CoV-2 S trimers: (1) nAb CC6.30, which targets the RBS-B or Class 2 epitope site, binds RBD with an affinity of 1.7 nM and directly competes with ACE2, and (2) nAb CC6.33, which targets the Class 3 epitope site that is distinct from the ACE2 binding site and binds RBD with an affinity of 257 nM (Figures 1 and 2). Several clinical-stage nAbs also recognize these epitopes such as CB6/LY-CoV16 (RBS-A or Class 1) (Shi et al., 2020; Yuan et al., 2021b), REGN10933 (RBS-B or Class 2) (Hansen et al., 2020; Yuan et al., 2021a) and S309 (Class 3) (Barnes et al., 2020; Pinto et al., 2020). In each complex, we used a stabilized, uncleaved Spike trimer (HPM7) based on the Wuhan strain containing 6 proline (hexaproline or HP) mutations (Hsieh et al., 2020) and an engineered interprotomer disulfide (mut7 or M7) between residues 705 and 883 of the S2 subunit. The global resolution of the two complexes was 3.6 Å (CC6.30/HPM7) and 3.3 Å (CC6.33/HPM7) and model building was assisted by local refinement maps of just the RBD and fragment antigen binding (Fab) variable region (Figures S1A, S1B, S2A, and S2B; Tables S1 and S2). In addition, we solved the ligand-free structure of the HPM7 spike (2.8 Å and 3.0 Å resolution for C3 symmetric and asymmetric, respectively) (Figures S2C and S2D; Table S2).

CC6.30 straddles the SARS-CoV-2 RBD ridge (approximately residues 473–490) with contributions from all heavy chain (HC) and light chain (LC) complementarity determining regions (CDRs) except for LCDR2 (Figures 1A and 1B). The HCDR3 contains a disulfide bond (C99-C100d, Kabat numbering) that makes it more rigid, perhaps in turn acting to stabilize this more flexible region of the RBD around the “ACE2-binding ridge” (approximately Spike residue numbers 470–490) (Figures S1B and S1C). Important interactions at the binding interface include hydrophobic packing of the RBD F490 side chain with antibody HC side chains I31, I52, I53 and I54 (Figure S1D), and predicted hydrogen bonding between the side chain of HCDR3 R97 and both the RBD I492 backbone carbonyl and Q493 side chain (Figure S1E). A salt bridge is formed between the side chains of RBD E484 and LCDR3 R96 (Figure 1C). This interaction is predicted to decrease the effectiveness of CC6.30 against certain variants, specifically Beta (Tegally et al., 2021) and Gamma (Faria et al., 2021) VOIs, which contain an E484K mutation that abolishes the salt bridge and causes a possible charge-charge repulsion. Of similar concern, the L452 side chain of the RBD has hydrophobic interactions with HCDR1 I31 and HCDR2 I53 which would be incompatible with a long, positively charged side chain that results from the L452R mutation found in the Delta variant (Planas et al., 2021) (Figure 1D).

CC6.30 appears to only bind the RBD-up state of the spike (Figure 1A). Although the ligand-free cryoEM structures of HPM7 spike reveal a 3 RBD-down conformation, our data suggest that the antibody is capable of shifting the equilibrium by capturing RBD in the up conformation. The most stable cryoEM reconstruction contains 2 up RBDs, each bound by CC6.30, whereas the unoccupied RBD remains in the down position (Figure 1A). Superposition of the RBD:CC6.30 portion of the model onto the all-down RBD ligand-free structure predicts that clashes would occur between HCDR3 and the RBD and N343 glycan from an adjacent protomer (Figure S1F). Finally, E484 is a major part of the epitope as defined in the Class 2 or RBS-B nomenclatures (Yuan et al., 2021a), consistent with our other observations for CC6.30.

CC6.33 binds a non-overlapping epitope to that of CC6.30, with the HC and LC interface centered on the N343 glycan (Figures 2A and 2B). In contrast to CC6.30, this nAb binds the RBD-down conformation and the most stable reconstruction has 2 down RBDs bound by the antibody, while the third RBD is in the up position (Figure 2A). Indeed, the binding of CC6.33 to a down RBD requires slight opening of the apex to relieve a clash with the RBD ridge of the adjacent protomer and is likely the driving force for the unoccupied RBD shifting to the up position after the binding of two Fabs (Figure S2E). Although modeling suggests that CC6.33 should be able to bind an up RBD, we did not observe this in our dataset, possibly because of the HPM7 spike design preferentially displaying 3 down-RBDs (Figure S2F). Also, portions of HC framework regions 1 (HFR1) and HFR3 contact the RBD ridge of the neighboring protomer (still in the down position) in a manner that mimics the interaction between the unoccupied up-RBD and an adjacent RBD-down ridge, further stabilizing the interaction between antibody and spike (Figures 2C and 2D). Binding of CC6.33 to its epitope is largely governed by hydrophobic interactions involving the heavy chain, including HCDR2 residues I52, I53 and L54 packing against RBD residues L335, V362 and P527 (Figure S2G). HCDR3 W98 reaches into an aromatic pocket lined with RBD residues F338, F342, A363, Y365 and L368, while also donating a hydrogen bond to the backbone carbonyl of D364 (Figure S2H). Fewer hydrogen bonds are predicted between RBD and CC6.33 compared to CC6.30. Those contributed by CC6.33 often involve bonds to RBD main chain atoms (e.g. HCDR3 Q97 with RBD backbone C336, V362 and D364), making such interactions less susceptible to changes in side chains resulting from VOC mutations (Figure S2I). The antibody epitope itself is largely positioned away from the common RBD mutations that could affect binding, a property shared with other Class 3 RBD antibodies (Figure 2D). Lastly, the light chain is mostly involved via LCDR2 packing against and providing hydrogen bonds to the viral N343 glycan, and a single peptide-peptide hydrogen bond between the side chains of LCDR1 Y32 and RBD E340 (Figures 2B and S2J).

### Engineering higher affinity SARS-CoV-2 antibodies

Affinity maturation of CC12.1, CC6.30 and CC6.33 was achieved using our rapid affinity maturation strategy, SAMPLER (Zhao et al., 2021). Briefly, HC and LC libraries were synthesized containing one mutation per CDR loop from the starting sequence, for up to three mutations per chain. Potential liabilities were informatically filtered from the library process and an N-linked glycan on LCDR1 of CC6.30 was removed by an N28S mutation, reverting that position to the original amino acid found in the germline VK1-39 gene segment so that any improved CC6.30 variant would not contain that glycan. The HC and LC libraries were displayed on the surface of yeast and iterative rounds of selections were used to enrich for clones with higher affinity for SARS-CoV-2 RBD (for CC12.1 and CC6.30 libraries) or S protein (for the CC6.33 library). The sort process also included a round of negative selection, where clones with low binding to a preparation of detergent-solubilized HEK293 cell membrane proteins were enriched to remove polyreactive variants. The enriched clones were then combined into a heavy/light combinatorial library and screened again with the same four-round selection strategy to identify the optimal heavy/light pairs (Zhao et al., 2021). At the conclusion of the selection process, sequences of the antibodies were recovered and 12 improved variants of each antibody were selected to be reformatted and expressed as IgG for characterization.

**Figure 3:**
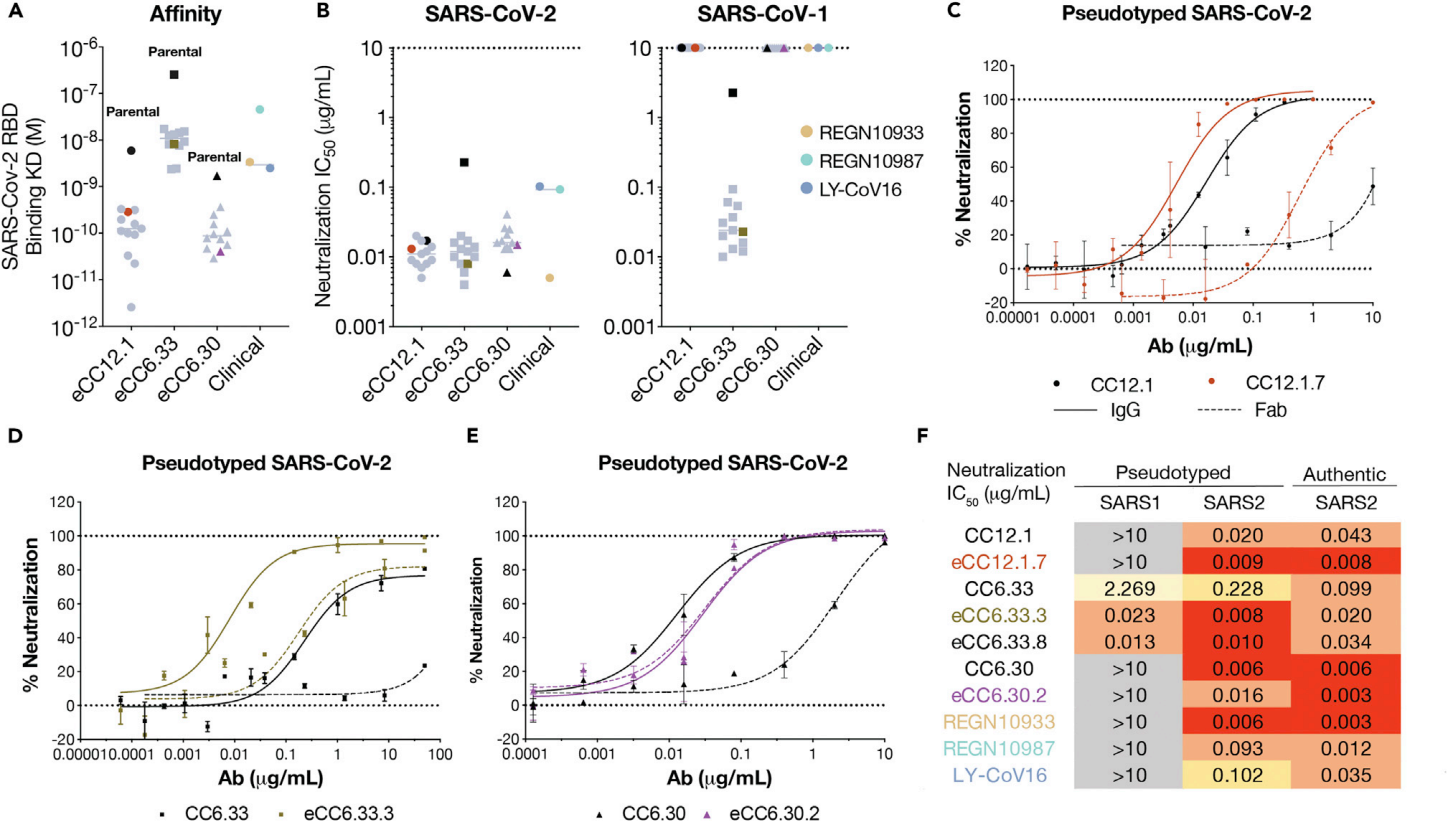
Binding affinity and neutralization potency of engineered SARS-CoV-2 nAbs (A) Enhanced and parental nAbs binding affinity against SARS-CoV-2 RBD by surface plasmon resonance. Parental nAbs are highlighted in black. eCC12.1.7, eCC6.33.3 and eCC6.30.2 are highlighted whereas other engineered variants are colored in grey. RBD binding to antibodies via a Fc-capture, multi-cycle method. Association and dissociation rate constants were calculated through a 1:1 Langmuir binding model using the BIAevaluation software. (B) Neutralization IC_50_ against pseudotyped SARS-CoV-2 and SARS-CoV-1 viruses. (C–E) SARS-CoV-2 pseudovirus neutralization curves of (C) parental CC12.1 and eCC12.1.7, (D) parental CC6.33 and eCC6.33.3, (E) parental CC6.30 and eCC6.30.2 in IgG and Fab molecules. Solid lines represent IgG neutralization whereas dashed lines represent Fab neutralization. Data are represented as mean ± SD. Data are representative of at least two independent experiments. (F) Summary table of nAb neutralization IC_50_ against pseudotyped SARS-CoV-1 and SARS-CoV2, as well as replicating SARS-CoV-2. See also Figures S3–S5; Table S3.

All enhanced (e) eCC12.1, eCC6.30, and eCC6.33 variants bound SARS-CoV-2 RBD with a higher affinity than the parental antibodies, with an average 45-fold (5- to 267-fold) increase in monovalent equilibrium dissociation constants (Figure 3A) measured by surface plasmon resonance (SPR). In nearly all cases, the affinity gains came through a reduction in the dissociation rate (Table S3). The binding affinity for monomeric RBD is notably lower for CC6.33 compared to CC12.1, CC6.30 (Figure 3A) and the majority of other antibodies isolated from our COVID-19 cohort (Rogers et al., 2020). The binding affinity of CC6.33 Fab for S protein is approximately 10-fold higher than for RBD, suggesting that the CC6.33 epitope is poorly formed on monomeric RBD and/or differential processing of the N343 glycan affects mAb binding. ELISA binding to SARS-CoV-2 RBD and S by CC12.1 and CC6.30 parental and engineered nAbs (enAbs) was comparable, however, a large improvement in neutralization EC_50_ and the maximum neutralization plateau was observed for eCC6.33 variants compared with the CC6.33 parental (Figure S3). The enAb variants were evaluated by analytical size exclusion chromatography and found to be monodispersed with similar column retention time to our clinical controls (Figure S4). None of the eCC12.1 or eCC6.33 variants bound to antigens in our polyreactivity panel (Chinese hamster ovary cell solubilized membrane proteins, single-stranded DNA, and human insulin) or stained HEp2 epithelial cells (Figure S5). Several of the CC6.30 variants showed low levels of binding to one or more of the antigens in our polyreactivity panel or stained HEp2 cells, but the majority of engineered variants were negative in all assays, highlighting the importance of expressing and validating multiple variants.

### Neutralizing activity of engineered antibodies

The parental and engineered nAbs were tested for *in vitro* neutralization of SARS-CoV-1 and SARS-CoV-2 pseudotyped viruses (Rogers et al., 2020) to investigate the relationship between improved binding affinity and *in vitro* neutralization potency. The results were different for the 3 antibodies. All eCC6.33 variants showed improved neutralization potency, with the IC_50_ improving from 228 ng/mL to around 10 ng/mL for SARS-CoV-2 (Figure 3B) and from 2.27 μg/mL to around 20 ng/mL for SARS-CoV-1. All eCC6.33 variants achieved 100% inhibition whereas the parental CC6.33 had a maximum percent neutralization (MPN) of only around 80% (Table S3). In stark contrast and despite comparable affinity improvements to eCC6.33 variants, eCC12.1 and eCC6.30 variants showed no significant improvement in neutralization potency relative to the parent Abs. To further investigate, we produced the parental and engineered nAbs as molecular Fabs to evaluate neutralization in a monovalent format. In all cases, the engineered Fabs neutralized more potently than the parental Fabs, with parental CC12.1 and CC6.33 Fabs failing to neutralize at concentrations of 50 μg/mL (Figures 3C–3E). As a control, we also tested our enAbs against authentic SARS-CoV-2 and observed similar neutralization activity to the pseudotyped virus (Figure 3F), consistent with our previous observations. Taken together, these data suggest that in this system increases in binding affinity translate to increases in the *in vitro* neutralization potency until a “threshold” IC_50_ around 10 ng/mL is reached, at which point further increases in binding affinity do not appear to affect the *in vitro* neutralization function of the antibody. Furthermore, this apparent affinity required to reach this neutralization threshold is lowered by the bivalent binding of an IgG. However, a Fab can neutralize the virus provided the monovalent affinity is sufficiently high, indicating inter- or intra- spike cross linking may help but is not necessary for these nAbs to neutralize (Figures 3C and 3E). It is also possible that larger IgG molecules more effectively obstruct access to the ACE2 receptor on target cells.

**Figure 4:**
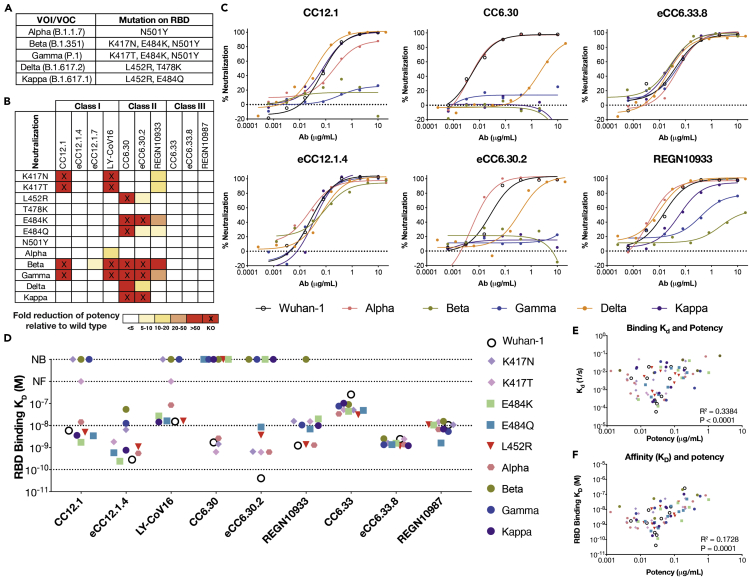
Antibody neutralization activities against circulating variants (A) Circulating SARS-CoV-2 variants of interest (Alpha, Beta, Gamma, Kappa) and variant of concern (Delta) and their mutations on RBD. (B) Summary table of fold reduction of neutralization potency of mAbs against circulating SARS-CoV-2 variants and single mutations relative to the original lineage Wuhan-1. Fold difference of neutralization potency was colored according to the key. (C) Representative neutralization curves of CC12.1, eCC12.1.7, CC6.30, eCC6.30.2, eCC6.33.8 and REGN10933 against Wuhan-1 as well as VOC including Alpha (B.1.1.7), Beta (B.1.351), Gamma (P.1), Delta (B.1.617.2), Kappa (B.1.617.1). Assays were run in duplicate. Data are representative of at least two independent experiments. SARS-CoV-2 and variants were colored according to the key. (D) Antibody binding affinity (K_D_) against wildtype SARS-CoV-2 RBD and RBD mutant proteins. NB: no binding. NF: Not fit to a simple kinetics model. Antibodies were captured to SPR sensors via a Fc-capture, multi-cycle method. Association and dissociation rate constants were calculated through a 1:1 Langmuir binding model using the BIAevaluation software. (E and F) Pearson correlation analysis between antibody (E) off-rate constant (Kd) or (F) equilibrium dissociation constant (K_D_) binding kinetics against RBD variants and antibody neutralization potency against mutant pseudoviruses. See also Figure S6 and Table S4.

We next sought to investigate how the evolving viral diversity of SARS-CoV-2 variants impacts the binding and the neutralization function of our parental and select engineered nAbs. We first measured the neutralization potency of our nAbs against VOI/sVOCs with full-spike mutations including Alpha (B.1.1.7, originating in UK), Beta (B.1.351, originating in South Africa) (Tegally et al., 2021), Gamma (P.1, originating in Brazil) (Faria et al., 2021), Kappa (B.1.617.1, originating in India) and Delta (B.1.617.2, originating in India) (Mlcochova et al., 2021; Planas et al., 2021) variants as well as single mutations on RBD (Figure 4A).

CC12.1 neutralized Alpha, Delta, and Kappa variants with an IC_50_ comparable to Wuhan-1 virus, however, Beta and Gamma VOIs completely escaped from this nAb (Figures 4B and 4C). Analysis of the individual variants found that K417N (from Beta VOI) and K417T (from Gamma VOI) facilitated this escape, consistent with the previous observation that most VH3-53-class nAbs are sensitive to these mutations (Francino-Urdaniz et al., 2021; Yuan et al., 2021a; Zhang et al., 2021a). K417 falls in the middle of the CC12.1 crystallographically defined epitope and makes hydrogen bonding or packing interactions with heavy chain residues H33, Y52, D95 and D97, as well as light chain residues N92 and K97 (Yuan et al., 2020). Importantly, eCC12.1.4 and eCC12.1.7 neutralized all VOCs, including Beta and Delta, containing the K417N/T mutations (Figure 4C). We measured neutralization of all 12 eCC12.1 antibodies against Beta and Gamma as well as the single mutation variants, where we found 11 out of 12 mAbs neutralized the Gamma lineage and 9 out of 12 neutralized the Beta lineage (Figure S6A). Mutational analysis of the 9 enAbs that reacted against both VOIs found a broad assortment of mutations that had been selected across the different antibodies (Figure S6A), suggesting that there are multiple ways to compensate for the loss of the K417 interaction.

CC6.30 and eCC6.30.2 were effective against the Alpha variant, showed significantly reduced function against the Delta variant, and were completely unable to neutralize the Beta, Gamma or Kappa variants (Figures 4B and 4C). When tested against pseudoviruses containing the individual mutations found in these variants, CC6.30 showed complete loss of neutralization against the single L452R, E484K, and E484Q variants. eCC6.30.2 retained modest functionality against L452R and E484Q, whereas the E484K variant still facilitated complete viral escape. This data was consistent with our structural analysis, with both E484 and L452 making extensive interactions with CC6.30 (Figures 1C and 1D).

The parental CC6.33 and eCC6.33.8 were effective at neutralizing all variants tested with similar IC_50_s to the original Wuhan-1 SARS-CoV-2, consistent with the observation that CC6.33 recognizes the conserved class 3 epitope (Barnes et al., 2020; Pinto et al., 2020) distal from the mutations in these viruses (Figures 2B, 4B, 4C, and S6B). REGN10987, another class 3 antibody (Barnes et al., 2020; Hansen et al., 2020) retained similar potency for all variants tested (Figure S6B).

To systematically investigate the relationship between binding affinity and neutralization for VOCs across our collection of parental and engineered nAbs, a panel of monomeric RBD variants were expressed for SPR analysis. Overall, the RBD binding affinities and the off-rate correlated well with the *in vitro* neutralization data (Figures 4D–4F). Mutations that completely abrogated neutralization usually showed a complete loss of binding by SPR or weak reactivity that could not be fit to a simple kinetics model. Of particular note were the Beta and Gamma RBDs binding to the CC12.1 variants. Parental CC12.1 bound Wuhan-1 RBD with an affinity of 6 nM and had a complete loss of both binding and neutralization to both Beta and Gamma RBDs. In contrast, affinity matured eCC12.1.4 bound Wuhan-1 with an affinity of 286 p.m., and although the mutations in Beta and Gamma reduced binding affinity by 182-fold and 43- fold (Table S4), respectively, eCC12.1.4 was still able to neutralize both VOIs (Figure 4C). These data indicate that enhanced nAb affinity for the target antigen helps to offset the affinity losses resulting from viral mutations within the nAb paratope, allowing the nAb to maintain sufficient affinity for neutralization.

### Mapping RBD escape mutations for CC12.1 and eCC12.1.4

**Figure 5:**
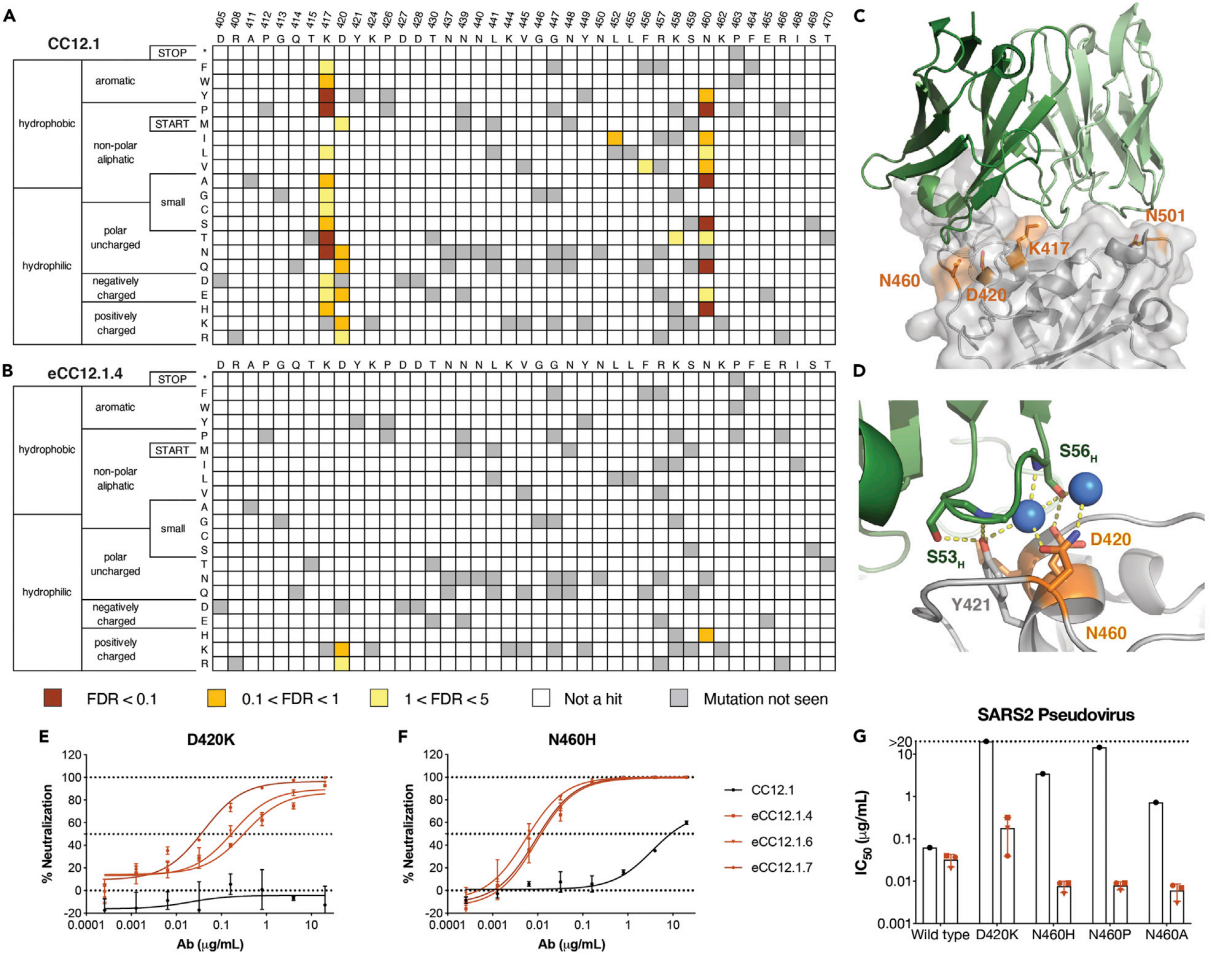
Mapping potential RBD viral escape mutants from CC12.1 and eCC12.1.4 (A and B) Putative viral escape screening of (A) CC12.1 and (B) eCC12.1.4 using a RBD yeast display platform (Francino-Urdaniz et al., 2021). RBD variants that did not disrupt ACE2 interaction but evaded nAb recognition were sorted and sequenced. A control with no ACE2 labeling was also sorted and served as an empirical false discovery rate (FDR). The enrichment ratio for each mutation relative to the reference population was colored according to the key. The heatmap of RBD residues 405–470 was shown, whereas the full map of residues from 333 to 527 was shown in Figure S7. (C and D) Crystal structure of CC12.1 interacting with SARS-CoV-2 RBD modified from PDB: 6XC2 (Yuan et al., 2020). CC12.1 heavy chain and light chain were colored in dark green and light green respectively. Key binding residues N501, K417, N460 and D420 on RBD were highlighted in orange. (E and F) Representative neutralization curves of CC12.1, eCC12.1, eCC12.1.6, and eCC12.1.7 against SARS-CoV-2 (E) D420K and (F) N460H. Antibodies were colored according to the key. Data were qwew represented as mean ± SD. Data were representative of at least two independent experiments. (G) Neutralization potency of parental CC12.1 and eCC12.1 variants against wildtype SARS-CoV-2 pseudovirus virus and D420K, N460H, N460P, N460A mutant viruses. See also Figure S7 and Table S5.

We next asked whether engineering high affinity nAbs reduces the pathways for viral escape compared with the parental nAbs or if they simply shifted the escape mutations to other positions. Deep mutational scanning libraries of RBD were generated and used to determine the mutations on RBD that prevented CC12.1 and eCC12.1.4 from blocking ACE2 binding *in vitro* (Figure S7A) (Francino-Urdaniz et al., 2021). 94.5% (2250/2380) of all possible single mutations were scanned (Table S5). Consistent with previous reports (Francino-Urdaniz et al., 2021) and our neutralization screening, CC12.1 is vulnerable to multiple mutations at K417 with a false discovery rate (FDR) below 0.1 for K417N/T but eCC12.1.4 is able to accommodate all mutations at K417 (Figures 5A, 5B, and S7B). We also detected multiple mutations at position D420 and N460 that confer escape from the parental CC12.1 (Figures 5A and 5B). Alanine scanning had identified these D420 and N460 residues as important for public VH3-53 SARS-CoV-2 antibodies (Yi et al., 2021; Zhang et al., 2021a), but structural analysis shows these two positions on the periphery of the CC12.1 epitope and making relatively insignificant contacts to the antibody (Figures 5C and 5D).

Pseudoviruses containing the individual D420K, N460H, N460P, and N460A mutations, identified as potential escape mutations in the deep mutational scanning, were produced and evaluated to determine if parental CC12.1 and several eCC12.1 variants were sensitive to these mutations in a neutralization assay. In agreement with RBD library screening, a D420K substitution completely disrupted CC12.1 neutralization, whereas substitutions at the N460 residue significantly decreased its neutralization potency by 12- to 246-fold (Figures 5E–5G). By contrast, although D420K and N460H were identified as potential escape mutations against eCC12.1.4, neutralization potency was reduced by a more modest 8-fold against D420K and remained insensitive to a N460H substitution (Figures 5D and 5F). These data suggest that increasing the affinity of SARS-CoV-2 nAbs restricts the potential escape mutations that can arise in RBD, rather than just altering the critical nAb contacts and shifting the escape mutations to a comparable number of different positions and/or mutations. This is particularly important in the context of developing antiviral antibodies where viral escape is a serious and constant threat.

### *In vivo* protection

**Figure 6:**
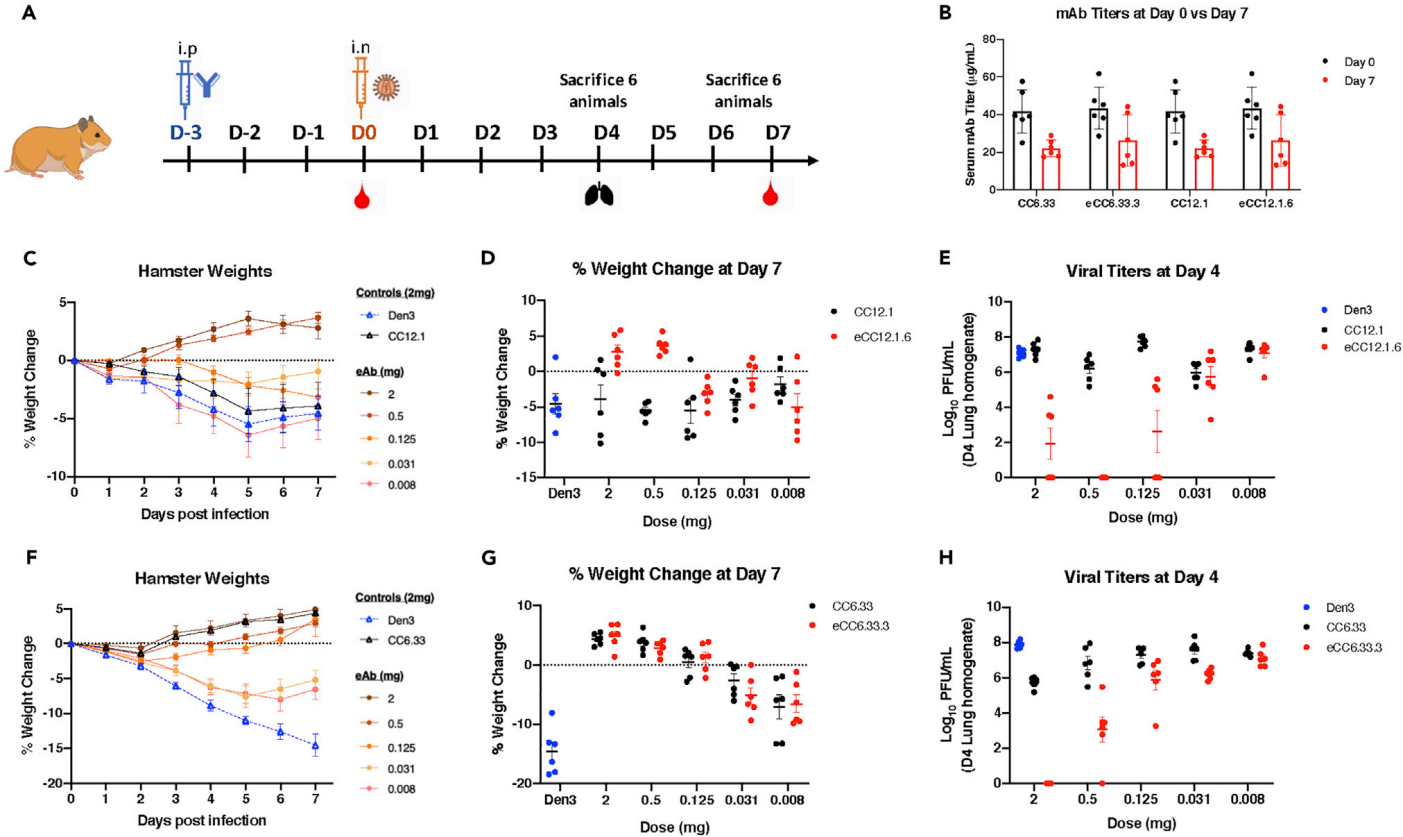
Protection of hamsters against SARS-CoV-2 challenge by parental and engineered nAbs (A) Overview of study design. Animal groups administered CC12.1 or eCC12.1.6 were challenged with 1 × 10^5^ PFU of SARS-CoV-2 (hCoV-19/South Africa/KRISP-EC-K005321/2020), those administered with CC6.33 or eCC6.33.3 were challenged with 1 × 10^5^ PFU of SARS-CoV-2 (USA-WA1/2020). (B) Serum mAb concentration at time of infection (Day 0) versus the time of sacrifice (Day 7). Data are represented as geometric mean ± SD. (C) Effect of CC12.1 vs eCC12.1.6 on weight loss in prophylaxis groups. (D) Percent weight change for all groups from (C) on day 7 after infection. Data are represented as geometric mean ± SD. (E) Viral load 4 days after infection as quantitated by live virus plaque assay on Vero E6 cells from lung tissue homogenate. Data are represented as geometric mean ± SD. (F) Effect of CC6.33 vs eCC6.33.3 on weight loss in prophylaxis groups. (G) Representative percent weight change for all groups from (F) on day 7 after infection. (H) viral load, detected as shown in (E). Statistical significance (p < 0.05) of groups in comparison to the Den3 IgG control group were calculated by Ordinary One-Way ANOVA test using Graph Pad Prism 8.0. Data are represented as geometric mean ± SD. See also Figures S8 and S9.

*In vitro* analysis of the enAbs suggested that the increased affinity provided functional improvements for two of the three candidates. eCC12.1 variants were better able to overcome mutations that are emerging in the VOCs and eCC6.33 variants had increased neutralization potency and higher MPN compared to the parental CC6.33. The improvements to CC6.30 were less clear and the parental antibody was found to have poor pharmacokinetics in hamsters and so was not pursued further *in vivo* (data not shown). A series of experiments were designed to compare parental and engineered nAbs in the Golden hamster model of COVID-19 infection. The experimental design for passive transfer studies is shown in Figure 6A. Groups of six hamsters were prophylactically treated with serially diluted doses of antibody starting at 2 mg per animal to 8 μg per animal via intraperitoneal (i.p.) injection 72 h before intranasal challenge with SARS-CoV-2 at a dose of 1 × 10^5^ plaque-forming units (PFU). A group receiving 2 mg doses of an irrelevant human mAb against dengue virus (Den3) was used as a control for each experiment. All hamsters were monitored daily for weight loss as a measure of disease (Roberts et al., 2005) and serum was collected from each animal to determine antibody titer at the time of viral challenge (D0) compared to the time of sacrifice (D7) (Figure 6B). Hamsters have been shown to clear SARS-CoV-2 infection after 7 days, so a replicate of the original experiment was performed in which the groups of hamsters were euthanized four days after infection (D4) and lung tissue was collected to quantify lung viral titers. In addition to collecting serum at D0 and D7 to measure nAb titers, the half-life of the parental and several engineered versions were assessed to try to find engineered nAbs that closely matched the bioavailability of the parental versions to allow a comparison of the two. Ultimately, eCC6.33.3 and eCC12.1.6 were selected to compare to the parental nAbs.

We first tested the ability of CC12.1 and eCC12.1.6 to protect against challenge from the Beta VOC, as the *in vitro* data showed that eCC12.1.6 effectively neutralized the variant (Figure S6B). The prophylactic protection experiment described above was done using Beta (20H/501Y.V2) SARS-CoV-2. Consistent with our *in vitro* neutralization data, eCC12.1.6 exhibited a dose-dependent protective response both in terms of weight loss and lung viral titers (Figures 6C–6E). Parental CC12.1 showed no protection compared to the Den3 control group. We also assessed eCC12.1.6 against the original SARS-CoV-2 (USA/WA1/2020) using the same groups (Figure S8) and the weight loss trend was nearly identical to that of the Beta variant, albeit the Beta variant showed a lower overall percentage of weight loss (Figure S8E).

The second protection experiment was designed to test whether the increased *in vitro* neutralization potency and maximum neutralization percentage of eCC6.33.3 relative to the parental nAb provided enhanced protection. CC6.33 (*in vitro* IC_50_ = 0.228 μg/mL and MPN of 81%) was compared with eCC6.33.3 (*in vitro* IC_50_ = 0.008 μg/mL and MPN of 100%) for protective efficacy against challenge from the original SARS-CoV-2 (USA/WA1/2020) (Figure S9). Following viral challenge, animals that received either the parental or engineered nAb, including the groups that received the 8 μg dose, showed a statistically significant reduction in weight loss compared to the group receiving the Den3 (Figures 6F and 6G). The protection from weight loss correlated with the amount of nAb the animals received, and there was no apparent difference in efficacy between the parental and engineered nAbs. However, in contrast to the weight loss results, the engineered nAbs showed superior ability to control viremia in the lung (Figure 6H). Animals that received the 2 mg dose of eCC6.33.3 had sterilizing immunity and the animals that received 500 μg dose of eCC6.33.3 had lung viral titers 5 logs lower than the Den3 control group. Animals that received the parental CC6.33 had viral titers only 2 logs lower than the Den3 group, protection that was comparable to the group receiving 125 μg of eCC6.33.3. It is unclear why there is a disconnect between lung viral titers and weight loss; however, eCC6.33.3 was clearly superior at controlling lung viral load. Broadly, the two experiments confirm that the affinity engineering of these nAbs provide a superior *in vivo* benefit predicted from *in vitro* analysis.

### Neutralization of the Omicron variant

During the preparation of this manuscript the Omicron VOC was reported. The variants characterized above contained 7 to 12 mutations in S compared to the original SARS-CoV-2, and at most contained 3 mutations in RBD (Beta and Gamma). In contrast, Omicron contains 30 mutations, 3 deletions and an insertion in S, with 15 of these mutations located in the RBD. The mutations in RBD are heavily concentrated across the class I, class II and class III neutralizing antibody epitopes and have been shown to reduce the neutralization efficacy of plasma from vaccinated and/or infected donors (Cameroni et al., 2022; Carreño et al., 2021; Dejnirattisai et al., 2021; Zhang et al., 2021b). They also confer resistance or complete escape from the majority of clinical antibody candidates (Cameroni et al., 2022).

**Figure 7:**
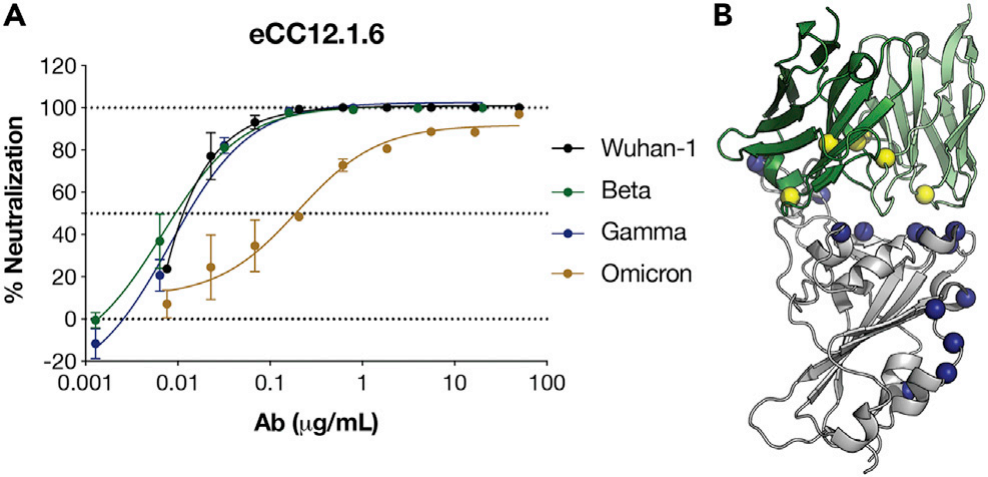
Neutralization and interaction of eCC12.1.6 against the Omicron variant (A) Representative neutralization curves of eCC12.1.6 against SARS-CoV-2 Beta, Gamma VOIs and Omicron VOC (B.1.1.519, GenBank: UIO84995.1). Wildtype SARS-CoV-2 and VOCs were colored according to the key. Data are represented as mean ± SD. (B) Structure of eCC12.1.6 interacting with Omicron RBD modified from PDB: 6XC2 (Yuan et al., 2020). eCC12.1.6 heavy chain and light chain were colored in dark green and light green respectively. Antibody mutant residues were highlighted in yellow spheres while Omicron RBD mutant residues were highlighted in dark blue spheres. See also Figure S10.

Omicron completely escaped from the parental CC6.30 and all eCC6.30 variants (Figure S10). This was largely consistent with data from other VOIs demonstrating the importance of the E484 interaction for this antibody family. Omicron not only contains an E484A mutation, but also contains a Q493R mutation that removes a hydrogen bond between Q493 and R97_H_ on CC6.30 variants. Similarly, the parental CC6.33 and all eCC6.33 variants were also unable to neutralize Omicron. This observation was more unexpected. The only Omicron mutation immediately within the CC6.33 epitope is G339D that may introduce a clash with the CC6.33 HCDR3 backbone. Omicron also contains mutations S371L, S373P and S375F that could alter the conformation of an adjacent loop and prevent the N343 glycan from adopting the conformation observed in the CC6.33 bound structure. Finally, we observed that Omicron was resistant to parental CC12.1 and 11 of 12 eCC12.1 variants, however, eCC12.1.6 retained neutralizing activity against Omicron (IC_50_ of 0.20 μg/mL), albeit with approximately 25-fold reduced potency (Figure 7A). eCC12.1.6 was also the most effective antibody against Beta and Gamma VOIs, with comparable potency to the Wuhan-1 strain (Figure 7A). Analysis of the selected mutations in eCC12.1.6 compared to other eCC12.1 variants did find a unique S31W mutation in LCDR2 that is located in close proximity to the N501Y mutation in all variants as well as G446S, G496S, Q498R and Y505H that are present in Omicron (Figure 7B). It is also possible that the other mutations in the antibody that do not interact directly with RBD stabilize the CDR loops in a conformation that happened to be slightly more compatible with Omicron compared to the other eCC12.1 variants.

## Discussion

The emerging SARS-CoV-2 variants have challenged vaccine-induced immunity and therapeutic mAbs, and functional nAbs with prophylactic and therapeutic efficacy against VOCs are desired. To this end, investigators have searched for nAbs that are largely resistant to viral antigenic variability by screening for nAbs that broadly neutralize SARS-CoV-2 variants and/or other sarbecoviruses. This effort has largely paralleled the work to identify broadly neutralizing HIV or influenza antibodies that target conserved epitopes on these antigenically variable viruses (Corti and Lanzavecchia, 2013; Sok and Burton, 2018). Here, we explored an alternative and complementary strategy of engineering nAbs to have higher affinity for their target antigen through directed evolution and then investigated the relationship between binding affinity, *in vitro* neutralization potency and *in vivo* efficacy. Broadly, we found that monovalent binding affinity and *in vitro* neutralization potency are correlated, until the *in vitro* neutralization IC_50_ reaches a “threshold” (around 10 ng/mL for IgG) after which further affinity improvements did not translate to improvements in neutralization potency, at least for CC12.1 and CC6.30 nAbs that directly compete with ACE2 binding to the RBD. These affinity improvements do help to expand the breadth of antibody reactivity, allowing them to better neutralize variants that contain mutations in and around the antibody epitope. This was particularly evident with the eCC12.1 nAbs that are part of the shared VH3-53 lineage and are broadly susceptible to the K417T/N mutations found in Beta and Gamma VOCs (Francino-Urdaniz et al., 2021; Yuan et al., 2021a). These mutations abrogated binding of the parental CC12.1 nAb, much like other reported VH3-53 nAbs, however, the higher affinity of the eCC12.1 variants for wild-type S-protein was able to compensate for the lost K417 contact, allowing the nAb to maintain sufficient affinity that it could still potently neutralize the VOIs. Of note, eCC12.1 variants were affinity matured against the original SARS-CoV-2 RBD sequence and had no specific selective pressure to accommodate mutations in the VOIs. Importantly, our saturated mutagenesis screening showed that the affinity maturation restricted the number of potential escape mutations rather than just altering them to different positions. Although increasing the affinity can restrict escape mutations, it does not abrogate them entirely, as eCC12.1 variants showed modest sensitivity to the D420K mutation, and all eCC6.30 variants were still unable to bind or neutralize the E484K mutation found in Beta and Gamma VOIs. In the extreme case of the Omicron variant that contains so many mutations on the RBD, especially within the footprint of class 1 antibodies, there was still one eCC12.1 variant that had significant neutralizing activity. This finding illustrates the value of affinity maturation in the context of natural infection where the generation of a diverse set of related antibodies, as was done here *in vitro*, will likely generate some antibodies able to bind to and act against many different viral variants, including those with multiple mutations as for Omicron. It is possible that part of the reason so many clinical antibody candidates failed against Omicron is that most were selected shortly after COVID infection before much affinity maturation had occurred (Liu et al., 2021).

The engineering of higher affinity enAbs also improved protective efficacy *in vivo*. The protection datashow that eCC12.1.6 was effective at preventing infection from the Beta variant whereas CC12.1 was not, consistent with the *in vitro* neutralization results. eCC6.33.3 provided significantly enhanced viral control with sterilizing immunity at the 2 mg dose and ∼100,000 fold reduction in lung viral titers at the 500 μg dose compared to the Den3 control. A plausible explanation for this enhanced control is the improvement in neutralization plateau—CC6.33 only achieves ∼80% MPN whereas eCC6.33 variants plateau at 100% neutralization. Although the results were expected, it was important to formally demonstrate that the affinity/neutralization improvements translated to enhanced protection *in vivo*. We also note that although not the objective of the experiment, the engineering produces a collection of related nAbs with different biochemical properties and this resulted in an eCC6.30 variant with significantly better pharmacokinetics compared to the parental CC6.30. Taken together, this data strongly suggests the benefit of engineering anti-viral nAbs for improved affinity to their target antigen, with an important benefit being the restriction of potential escape pathways for the virus.

The data also emphasizes the importance of affinity maturation in vaccine-induced responses to maximize the opportunities for protection against viral variants. Longitudinal studies have shown that mAbs isolated from later time points have more somatic mutation compared to clonally related variants from earlier time points, and this additional somatic mutation can increase affinity, potency and expand breadth (Gaebler et al., 2021; Muecksch et al., 2021). The enAb selections demonstrated that there are numerous solutions from a single starting point that can produce antibodies with increased binding affinity (Table S3). This situation is similar to a vaccine-expanded B cell lineage that undergoes slightly different maturation trajectories. Despite achieving similar results against the target antigen, the different members within the expanded lineage can have different levels of cross-reactivity to viral variants. We also note that the binding kinetics of our enAbs are consistent with what can be achieved through multiple vaccinations with a soluble protein antigen (Poulsen et al., 2011). These data support the current approach of boosting with the original SARS-CoV-2 vaccine formulation, as continued expansion of B cell lineages and further improvements in affinity can help expand the response to cover new VOCs. It also will help to expand the pool of memory B cells that may be cross-reactive to new vaccine formulations when they are eventually developed.

### Limitations of the study

All Abs in this study were expressed as human IgG1. In hamsters, it is not well understood if/how a human Fc interacts with hamster Fc receptors, thus the observed protection is likely only attributed to viral neutralization. Similarly, Fc functions are not evaluated in the *in vitro* neutralization assays. We were also unable to provide a mechanism for the neutralization threshold that seems to appear when the IC_50_ reaches the 1–10 ng/mL range. We have observed this effect in both SARS-CoV-2 and HIV pseudovirus assays and hypothesize that it could be because of Ab depletion in the assay.

## STAR★Methods

### Key resources table

**Table:** 

REAGENT or RESOURCE	SOURCE	IDENTIFIER
**Antibodies**		

FITC chicken anti-C-Myc antibody	Immunology Consultants Laboratory	Cat#CMYC-45F
AF405 anti-V5 antibody	IAVI/TSRI	N/A
Streptavidin-APC	Invitrogen	Cat#SA1005
6× His tag monoclonal antibody	Invitrogen	Cat#MA1-21315; RRID:AB_557403
Alkaline phosphatase-conjugated goat anti-human Fcγ antibody	Jackson ImmunoResearch	Cat#109-055-190; RRID:AB_2888997
Peroxidase goat anti-Human IgG (H+L) secondary antibody	Jackson ImmunoResearch	Cat#109-035-088; RRID:AB_2337584
CC12.1	(Rogers et al., 2020)	N/A
CC6.33	(Rogers et al., 2020)	N/A
CC6.30	(Rogers et al., 2020)	N/A
REGN10933	(Hansen et al., 2020)	N/A
REGN10987	(Hansen et al., 2020)	N/A
LY-CoV16	(Shi et al., 2020)	N/A
eCC12.1.1-eCC12.1.12	This paper	k: /
eCC6.33.1-eCC6.33.12	This paper	Genbank: ON585728 - ON585739; ON585763 - ON585774
eCC6.30.1-eCC6.30.11	This paper	Genbank: ON585740 - ON585750; ON585775 - ON585785

**Bacterial and virus strains**		

SARS-CoV-2 (USA-WA1/2020)	(Rogers et al., 2020)	N/A
SARS-CoV-2 B.1.351 (20H/501Y.V2)	This paper	N/A

**Chemicals, peptides, and recombinant proteins**		

Synthetic drop-out medium without tryptophan	Sunrise	Cat#1709-500
Sodium valproic acid	Sigma-Aldrich	Cat#P4543
Zymolyase	Zymo Research	Cat#E1005
Phusion enzyme	ThermoFisher	Cat#F530L
45% D-(+)- glucose	Sigma-Aldrich	Cat#G8769-100ML
Human insulin	Sigma-Aldrich	Cat#I2643
Lipofectamine 2000	ThermoFisher	Cat#11668027
FectoPRO	Polyplus	Cat#116-010
Protein A sepharose	GE Healthcare	Cat#17-5280-04
HisPur Ni-NTA Resin	ThermoFisher	Cat#88222
Alkaline phosphatase substrate	Sigma-Aldrich	Cat#S0942-200TAB
HRP substrate	Roche	Cat#11582950001
Fetal Bovine Serum (FBS)	Omega Scientific	Cat#FB-02
L-Glutamine	Corning	Cat#25-005-Cl
Penicillin/Streptomycin	Corning	Cat#30-002-C
BrightGlo substrate	Promega	Cat#PR-E2620
n-dodecyl-β-D-maltopyranoside (DDM)	Anatrace	Cat#D310

**Critical commercial assays**		

Pierce Fab preparation kit	ThermoFisher	Cat#44985
CaptureSelect CH1-XL Affinity Matrix	ThermoFisher	Cat#2943452010
Expi293 Expression System Kit	ThermoFisher	Cat#A14635
ExpiCHO Expression System Kit	ThermoFisher	Cat#A29133
FreeStyle 293 expression medium	Gibco	Cat#12338002
Opti-MEM medium	Gibco	Cat#51985091
DMEM medium	Corning	Cat#15-013-CV
HEp2 slides	Hemagen	Cat#902360

**Deposited data**		

CC6.30 Fab + SARS-CoV-2 S HPM7 (non-uniform refinement)	This paper	EMD-24697; PDB 7ru5
CC6.30 Fab + SARS-CoV-2 S HPM7 (RBD/Fv local refinement)	This paper	EMD-24699; PDB 7ru8
CC6.33 IgG + SARS-CoV-2 S HPM7 (non-uniform refinement)	This paper	EMD-24695; PDB 7ru3
CC6.33 IgG + SARS-CoV-2 S HPM7 (local refinement)	This paper	EMD-24696; PDB 7ru4
SARS-CoV-2 S HPM7 (C3 symmetry)	This paper	EMD-24693; PDB 7ru1
SARS-CoV-2 S HPM7 (C1 symmetry)	This paper	EMD-24694; PDB 7ru2

**Experimental models: Cell lines**		

HEK293T cells	ATCC	Cat#CRL-3216; RRID: CVCL_0063
FreeStyle HEK293F cells	ThermoFisher	Cat#R79007; RRID: CVCL_D603
Expi293F cells	ThermoFisher	Cat#A14527; RRID: CVCL_D615
YVH10 cells	ATCC	Cat#MYA4940
Vero-E6 cells	ATCC	Cat#CRL-1586; RRID: CVCL_0574
EBY100 cells	ATCC	Cat#MYA4941
Hela-hACE2 cells	(Rogers et al., 2020)	N/A

**Experimental models: Organisms/strains**		

Golden Syrian hamsters	Charles River	N/A

**Recombinant DNA**		

pYDSI yeast vector	(Zhao et al., 2021)	N/A
MLV Gag/Pol backbone	Addgene	Cat#14887
MLV-CMV-Luciferase plasmid	Addgene	Cat#170575
SARS-CoV-2-d18 spike plasmid	(Rogers et al., 2020)	N/A
SARS-CoV-1-d28 spike plasmid	(Rogers et al., 2020)	N/A
B.1.1.7-d18 spike plasmid	This paper	N/A
B.1.351-d18 spike plasmid	This paper	N/A
P.1-d18 spike plasmid	This paper	N/A
B.1.617.1-d18 spike plasmid	This paper	N/A
B.1.617.2-d18 spike plasmid	This paper	N/A
SARS-CoV-2 S HPM7 spike plasmid	(Yuan et al., 2021)	N/A

**Software and algorithms**		

Prism v8.0	GraphPad	https://www.graphpad.com/scientific-software/prism/
MotionCorr2	(Zheng et al., 2017)	https://emcore.ucsf.edu/ucsf-software; RRID:SCR_016499
cryoSPARC	(Punjani et al., 2017)	https://cryosparc.com/; RRID:SCR_016501
Relion 3.1	(Zivanov et al., 2020)	https://www2.mrc-lmb.cam.ac.uk/relion; RRID:SCR_016274
ABody Builder	(Leem et al., 2016)	http://opig.stats.ox.ac.uk/webapps/newsabdab/sabpred/abodybuilder/
UCSF Chimera	(Pettersen et al., 2004)	http://plato.cgl.ucsf.edu/chimera/; RRID:SCR_004097
Coot 0.9	(Casañal et al., 2020)	https://www2.mrc-lmb.cam.ac.uk/personal/pemsley/coot/; RRID:SCR_014222
Rosetta	(Conway et al., 2014)	https://www.rosettacommons.org; RRID:SCR_015701
EMRinger	(Barad et al., 2015)	https://www.phenix-online.org
Molprobity	(Williams et al., 2018)	https://www.phenix-online.org/; RRID:SCR_014226
Phenix software suite	(Liebschner et al., 2019)	https://www.phenix-online.org

### Resource availability

#### Lead contact

Further information and requests for resources and reagents should be directed to and will be fulfilled by the Lead Contact, Joseph G. Jardine (jjardine@iavi.org).

#### Materials availability

• Antibody plasmids and protein plasmids in this study are available with a material transfer agreement (MTA)
• Pseudovirus mutant plasmids in this study are available with a material transfer agreement (MTA)

### Experimental model and subject details

#### Syrian hamsters

Golden Syrian hamsters were provided by Charles River Laboratories (CRL:LVG(SYR)) and housed at the Scripps Research Institute. Male 12–13-week-old hamsters were infused with antibodies intraperitoneally as described previously (Rogers et al., 2020). The Scripps Research Institutional Animal Care and Use Committee (IACUC) approved all experimental procedures involving all the animals in accordance with Protocol #20-0003.

#### Cell lines

*Saccharomyces cerevisiae* YVH10 cells (ATCC) were used in antibody library generation and FACS sort. Hela-hACE2 cells (Rogers et al., 2020) were used in pseudovirus neutralization assay. *Saccharomyces cerevisiae* EBY100 cells (ATCC) were used in RBD mutant library generation and FACS sort. Human HEK293T cells (ATCC) were used for pseudovirus production. FreeStyle HEK293 cells (ThermoFisher) were used for recombinant S protein production. Expi293F cells (ThermoFisher) were used for monoclonal antibody and recombinant RBD production. Vero-E6 cells (ATCC) were used for live virus plaque assay.

### Method details

#### Recombinant S and RBD production

SARS-CoV-1 (Genbank: AAP13567) or SARS-CoV-2 (Genbank: MN908947) S proteins were transiently expressed in Freestyle 293F system (ThermoFisher) whereas RBD proteins were expressed in the Expi293 system (ThermoFisher). In brief, S expression plasmids were cotransfected with 40K PEI (1 mg/mL) at a ratio of 1:3. After incubation for 30 min at RT, transfection mixture was added to Freestyle 293F cells at a density of approximately 1 million cells/mL. RBD plasmids with His-Avitag were cotransfected with FectoPRO (Polyplus 116-010). SARS-CoV-2 RBD mutant plasmids were generated by Quikchange site-directed mutagenesis according to manufacturer’s instructions (Agilent, 210513). Biotinylated proteins were made by co-transfecting Avitagged RBD plasmids with a BirA expression plasmid and into Expi293 cells using FectoPRO. After incubation for 10 min at RT, transfection mixture was added to Expi293 cells at a density of ∼ 3 million cells/mL. After 24h of transfection, cells were fed with D-(+)- glucose solution and 300 mM of sterile sodium valproic acid solution. After 5 days of transfection, supernatants were harvested and filtered with a 0.22 μm membrane. The His-tagged proteins were purified with the HisPur Ni-NTA Resin (Thermo Fisher, 88222). After three columns of washing with 25 mM Imidazole (pH 7.4), proteins were eluted with an elution buffer (250 mM Imidazole, pH 7.4) at slow gravity speed (∼4 s/drop). Eluted proteins were buffer exchanged and concentrated with PBS using Amicon tubes (Millipore). The proteins were further purified by size exclusion chromatography (SEC) using Superdex 200 (GE Healthcare). The selected fractions were pooled and concentrated.

For cryoEM, a stabilized version of CoV-2 S protein, HPM7 (hexaproline mutant 7) was used. The design, expression and purification has been described previously (Yuan et al., 2021a). Briefly, the S protein is stabilized with 6 engineered proline residues (first described in (Hsieh et al., 2020)) and an interprotomer disulfide between residues 705 and 883 of S2. HPM7 was expressed in HEK293F, and purified using a C-terminal 2× StrepTag followed by size exclusion chromatography to isolate trimers.

#### Antibody production and purification

Monoclonal antibody was transiently expressed in the Expi293 system (ThermoFisher, A14635). In brief, antibody HC and LC plasmids were co-transfected at a ratio of 1:2.5 with transfection reagent FectoPRO (Polyplus 116-010). After 24 h of transfection, 300 mM of sterile sodium valproic acid solution (Sigma-Aldrich, P4543) and 45% D-(+)- glucose solution (Sigma Aldrich, G8769-100ML) were added to feed cells. After 4–5 days of transfection, supernatants were collected, sterile-filtered (0.22 μm), and IgG was purified with Protein A sepharose beads (GE Healthcare 17-5280-04).

#### Expression and purification of Fab

The CC6.33, eCC6.33.3, CC6.30, eCC6.30.2 Fabs were purified by digesting IgG using Fab digestion kit (ThermoFisher, 44985) according to manufacturer’s instructions. After digestion, Fc fragments and undigested IgG were removed from binding to the protein A beads (GE Healthcare). CC12.1 and eCC12.1.7 Fab fragments were generated by introducing a stop codon after the CH1 region in heavy chain expression plasmids. After transfection, Fabs were purified using Capto L beads (Cytiva, 17547801).

#### CryoEM data collection and processing

Toform the antibody-spike complexes, CC6.30 Fab or CC6.33 IgG was incubated with HPM7 spike trimer for approximately 15 min. A 9:2 molar ratio of CC6.30 Fab to spike or 1:2 molar ratio of CC6.33 IgG to spike was used. Samples were concentrated to about 1.7 mg/mL, n-dodecyl-β-D-maltopyranoside (DDM) was added to a final concentration of 0.06 mM, and the mixture and applied to plasma cleaned Quantifoil 1.2/1.3 400 copper mesh, holey carbon grids. Grids were vitrified using the Vitrobot Mark IV system (Thermo Fisher) set to 4°C, 100% humidity, with a blot time of 3 s.

Data were collected on a Thermo Fisher Titan Krios equipped with a Gatan K2 Summit direct electron detector. Detailed data collection statistics are summarized in Tables S1 and S2. Movie micrographs were motion corrected and dose weighted using MotionCorr2 (Zheng et al., 2017). Aligned micrographs were imported into cryoSPARC version 3.2 (Punjani et al., 2017) and CTF corrections were performed using the Patch CTF application in cryoSPARC. Automated particle picking, particle extraction, and initial 2D classifications were performed in cryoSPARC. Particles belonging to selected 2D classes were then imported into Relion 3.1 (Zivanov et al., 2020). Interactive rounds of 3D classification and refinement, and CTF refinements were performed for each dataset. To improve resolution of the antibody epitope and paratope, the best refinement from each dataset (Spike with 2 Fabs bound for both CC6.30 and CC6.33) was subjected to C3 symmetry expansion and focused classifications, using a spherical mask around the expected Fab/RBD region of a single protomer and Relion 3D classification without alignments. Particles containing density for Fab and RBD in the region of interest were imported into cryoSPARC. Signal outside of the RBD and Fab Fv was subtracted, and the subtracted particles were subjected to cryoSPARC local refinement (Figures S1A, S1B, S2A, and S2B). The non-symmetry expanded particles from the best Relion global refinements were imported into cryoSPARC and subjected to a final round of non-uniform refinement (Punjani et al., 2020). Additionally, the CC6.33 dataset contained thousands of ligand-free HMP7 Spike particles which were also imported into cryoSPARC for final non-uniform refinement, with or without symmetry (Figures S2C and S2D). Final Fourier shell correlation resolution estimates for all maps, along with EMDB deposition codes, can be found in Tables S1 and S2.

#### Cryo-EM model building

Homology models of the Fab variable regions of CC6.30 and CC6.33 were generated using ABodyBuilder (Leem et al., 2016) and fitted into the respective local refinement maps using UCSF Chimera (Pettersen et al., 2004). Coordinates for RBD with complete ridge were taken from PDB 7byr. The RBD:Fv models were subjected to interactive cycles of manual and automated refinement using Coot 0.9 (Casañal et al., 2020) and Rosetta (Conway et al., 2014). Once a high map-to-model agreement was reaching, as measured by EMRinger (Barad et al., 2015), and geometries were optimized, as judged by MolProbity (Williams et al., 2018), the models were fit into the non-uniform refinement full trimer maps and combined with a Spike model refined into the ligand-free map (PDB 6vxx was used as the initial model and HPM7 mutations were added manually in Coot following iterative rounds of Rosetta relaxed refinement and Coot manual editing). The resulting Fv:trimer models were refined in Rosetta. The Phenix software suite (Liebschner et al., 2019) was used for structure validation, and for editing and preparation of PDB files for deposition. Final refinement statistics and PDB deposition codes for generated models can be found in Tables S1 and S2.

#### Antibody library generation

CC12.1, CC6.30 and CC6.33 heavy chain and light chain affinity maturation libraries were synthesized as Oligopools (Integrated DNA Technologies). Mutations were included in the CDR loops and the CDR1/2/3 mini-libraries were assembled into combinatorial heavy chain and light chain libraries as previously described (Zhao et al., 2021). The libraries were displayed on the surface of yeast as molecular Fab using the yeast display vector pYDSI containing the bidirectional Gal1-10 promoter. The heavy chain contains a C-terminal V5 epitope tag and the light chain contains a C-terminal C-myc epitope tag to assess the amount of Fab displayed on the surface of the yeast. The HC library was generated by cloning the HC CDR1/2/3 library into the vector containing the wildtype light chain by homologous recombination, and the LC library was generated by doing the inverse. The combinatorial H/L library was generated by amplifying the HC and LC sequences with primers overlapping in the Gal1-10 promoter. The recovered Gal-HC and Gal-LC fragments were ligated via Gibson assembly and amplified. The resulting LC-Gal1-10-HC product was cloned into an empty pYDSI vector by homologous recombination (Zhao et al., 2021).

#### Yeast library labeling and sorting

After yeast transformation, yeast cells were passaged 1:20 the following day. Cells were then induced at OD_600_ = 1.0 overnight at 30°C in SGCAA induction medium (20 g galactose, 1 g glucose, 6.7 g yeast nitrogen base without amino acid, 5 g bacto casamino acids, 5.4 g Na_2_HPO_4_, 8.56 g NaH_2_PO_4_⋅H_2_O, 8.56 mg uracil in 1 L deionized water, pH 6.5, and sterilize by filtration). For each library, in the first round of selection, 5 × 10^7^ of yeast cells were stained per sample. In the second to final round of selection, 1 × 10^7^ cells were stained. Yeast cells were firstly spun down and washed with PBSA (PBS + 1% BSA), then incubated with biotinylated SARS-CoV-2 RBD or S or HEK cell membrane protein at several non-depleting concentrations respectively for at least 30 min at 4°C. After washing, yeast cells were stained with FITC-conjugated chicken anti-C-Myc antibody (Immunology Consultants Laboratory, CMYC-45F), AF405-conjugated anti-V5 antibody (made in house), and streptavidin-APC (Invitrogen, SA1005) in 1:100 dilution for 20 min at 4°C. After washing, yeast cells were resuspended in 1 mL of PBSA and loaded on BD FACSMelody cell sorter. Top 5–10% of cells with high binding activity to a certain SARS-CoV-2 RBD labeling concentration were sorted and spun down. Sorted cells were expanded in 2 mL of synthetic drop-out medium without tryptophan (Sunrise, 1709-500) supplemented with 1% Penicillin/Streptomycin (Corning, 30-002-C) at 30°C overnight.

#### Size exclusion chromatography analysis

The antibodies were analyzed by size exclusion chromatography using the 1260 Infinity II (Agilent). 15 uL of each antibody at 2 mg/mL was injected into the TSKgel SuperSW mAb column (Tosoh) with the flow rate of 1 mL/min.

#### Pseudovirus neutralization assay

Pseudovirus was generated as described previously (Rogers et al., 2020). In brief, MLV gag/pol backbone (Addgene, 14,887), MLV-CMV-Luciferase plasmid (Addgene, 170575), and SARS-CoV-2-d18 (Genbank: MN908947) or SARS-CoV-1-d28 (Genbank: AAP13567) or SARS-CoV-2 VOC spike plasmid were incubated with transfection reagent Lipofectamine 2000 (Thermo Fisher, 11668027) following manufacturer’s instructions for 20 min at RT. Full-spike mutations were introduced by overlapping extension polymerase chain reaction (PCR) to generate mutated spikes of circulating SARS-CoV-2 VOI/VOC, i.e., Alpha, Beta, Gamma, Delta, Kappa, and Omicron. Then the mixture was transferred onto HEK 293T cells (ATCC, CRL-3216) in a 10 cm^2^ culture dish (Corning, 430293). After 12–16 h of transfection, culture medium was gently removed, fresh DMEM medium was added onto the culture dish. Supernatants containing pseudovirus were harvested after 48 h post transfection and frozen at −80°C for long term storage.

In the neutralization assay, antibody samples were serially diluted with complete DMEM medium (Corning, 15-013-CV) containing 10% FBS (Omega Scientific, FB-02), 2 mM L-Glutamine (Corning, 25-005-Cl), and 100 U/mL of Penicillin/Streptomycin (Corning, 30-002-C). 25 μL/well of diluted samples were then incubated with 25 μL/well of pseudotyped virus for 1hat 37°C in 96-well half-area plates (Corning, 3688). After that, 50 μL of Hela-hACE2 cells at 10,000 cells/well with 20 μg/mL of Dextran were added onto each well of the plates. After 48 h of incubation, cell culture medium was removed, luciferase lysis buffer (25 mM Gly-Gly pH 7.8, 15 mM MgSO4, 4 mM EGTA, 1% Triton X-100) was added onto cells. Luciferase activity was measured by BrightGlo substrate (Promega, PR-E2620) according to the manufacturer’s instructions. mAbs were tested in duplicate wells and independently repeated at least twice. Neutralization IC_50_ values were calculated using “One-Site Fit LogIC50” regression in GraphPad Prism 8.0.

#### Authentic SARS-CoV-2 neutralization assay

Vero-E6 cells were seeded in 96-well half-well plates at approximately 8000 cells/well in a total volume of 50 μL complete DMEM medium the day prior to the addition of antibody and virus mixture. The virus (500 plaque forming units/well) and antibodies were mixed, incubated for 30 min and added to the cells. The transduced cells were incubated at 37°C for 24 h. Each treatment was tested in duplicate. The medium was removed and disposed of appropriately. Cells were fixed by immersing the plate into 4% formaldehyde for 1 h before washing 3 times with phosphate buffered saline (PBS). The plate was then either stored at 4°C or gently shaken for 30 min with 100 μL/well of permeabilization buffer (PBS with 1% Triton-X). All solutions were removed, then 100 μL of 3% bovine serum albumin (BSA) was added, followed by room temperature (RT) incubation at 2 h.

A mix of primary antibodies consisting of CC6.29, CC6.33, CC6.36, CC12.23, CC12.25 (Rogers et al., 2020) in equal amounts for detection. The primary antibody mixture was diluted in PBS/1% BSA to a final concentration of 2 μg/mL. The blocking solution was removed and washed thoroughly with wash buffer (PBS with 0.1% Tween 20). The primary antibody mixture, 50 μL/well, was incubated with the cells for 2 h at RT. The plates were washed 3 times with wash buffer.

Peroxidase AffiniPure Goat Anti-Human IgG (H+L) secondary antibody (Jackson ImmunoResearch, 109-035-088) diluted to 0.5 μg/mLl in PBS/1% BSA was added at 50 μL/well and incubated for 2 h at RT. The plates were washed 6 times with wash buffer. HRP substrate (Roche, 11582950001) was freshly prepared as follows: Solution A was added to Solution B in a 100:1 ratio and stirred for 15 min at RT. The substrate was added at 50 μL/well and chemiluminescence was measured in a microplate luminescence reader (BioTek, Synergy 2).

The following method was used to calculate the percentage neutralization of SARS-CoV-2. First, we plotted a standard curve of serially diluted virus (3000, 1000, 333, 111, 37, 12, 4, 1 PFU) versus RLU using four-parameter logistic regression (GraphPad Prism 8.0).

#### Recombinant protein ELISA

6x-His tag antibodies (Invitrogen, MA1-21315) were coated at 2 μg/mL in PBS onto 96-well half-area high binding plates (Corning, 3690) overnight at 4°C. After washing and blocking, 1 μg/mL of his tagged recombinant SARS-CoV-2 (or SARS-CoV-1) RBD or S proteins were diluted in PBS with 1% BSA and incubated for 1hat RT. After washing, serially diluted antibodies were added in plates and incubated for 1hat RT. After washing, alkaline phosphatase-conjugated goat anti-human IgG Fcγ secondary antibody (Jackson ImmunoResearch, 109-055-008) was added in 1:1000 dilution and incubated for 1hat RT. After final wash, phosphatase substrate (Sigma-Aldrich, S0942-200TAB) was added into each well. Absorption was measured at 405 nm.

#### Polyspecificity reagent (PSR) ELISA

Solubilized CHO cell membrane proteins (SMP) were made in house. SMP, human insulin (Sigma-Aldrich, I2643), single strand DNA (Sigma-Aldrich, D8899) were coated onto 96-well half-area high-binding ELISA plates (Corning, 3690) at 5 μg/mL in PBS overnight at 4°C. After washing, plates were blocked with PBS/3% BSA for 1 h at RT. Antibody samples were diluted at 100 μg/mL in 1% BSA with serial dilution and then added in plates and incubated for 1 h at RT. After washing, alkaline phosphatase-conjugated goat anti-human IgG Fcγ secondary antibody (Jackson ImmunoResearch, 109-055-008) was added in 1:1000 dilution and incubated for 1h at RT. After final wash, phosphatase substrate (Sigma-Aldrich, S0942-200TAB) was added into each well. Absorption was measured at 405 nm.

#### HEp2 epithelial cell polyreactive assay

Reactivity to human epithelial type 2 (HEp2) cells was determined by indirect immunofluorescence on HEp2 slides (Hemagen, 902360) according to manufacturer’s instructions. Briefly, mAbs were diluted at 100 μg/mL in PBS respectively and then incubated onto immobilized HEp2 slides for 30 min at RT. After washing, one drop of FITC-conjugated goat anti-human IgG antibody was added onto each well and incubated in the dark for 15–30 min at RT. After washing with PBS, a cover slide was added to HEp2 cells with glycerol and the slide was photographed on a Nikon fluorescence microscope to detect GFP. All panels were shown at magnification 40×.

#### Surface plasmon resonance methods

SPR measurements were carried out on a Biacore 8K instrument at 25°C. All experiments were carried out with a flow rate of 30 μL/min in a mobile phase of HBS-EP [0.01 M HEPES (pH 7.4), 0.15 M NaCl, 3 mM EDTA, 0.0005% (v/v) Surfactant P20]. Anti-Human IgG (Fc) antibody (Cytiva) was immobilized to a density ∼7000–10000 RU via standard NHS/EDC coupling to a Series S CM-5 (Cytiva) sensor chip. A reference surface was generated through the same method.

For conventional kinetic/dose-response, listed antibodies were captured to 50–100 RU via Fc-capture on the active flow cell prior to analyte injection. A concentration series of SARS-CoV-2 RBD was injected across the antibody and control surface for 2 min, followed by a 5 min dissociation phase using a multi-cycle method. Regeneration of the surface in between injections of SARS-CoV-2 RBD was achieved by a single, 120s injection of 3M MgCl_2_. Kinetic analysis of each reference subtracted injection series was performed using the BIAEvaluation software (Cytiva). All sensorgram series were fit to a 1:1 (Langmuir) binding model of interaction.

#### RBD library generation and identification of escape mutants

Yeast display plasmids pJS697 and pJS699 used for surface display of Wuhan-Hu-1 S RBD N343Q were previously described (Banach et al., 2021). Using these plasmids, 119 surface exposed positions on the original Wuhan-Hu-1 S RBD N343Q (positions 333–537) were mutated to every other amino acid plus stop codon using degenerate NNK primers using comprehensive nicking mutagenesis (Wrenbeck et al., 2016) exactly as previously described (Francino-Urdaniz et al., 2021; Haas et al., 2021). Two tiles were constructed for compatibility with 250bp paired end Illumina sequencing (tile 1: positions 333–436; tile 2: positions 437–527). Libraries were transformed into *S. cerevisiae* EBY100 and stocks of 1e8 viable yeast cells in 1 mL were stored in yeast storage buffer (20 w/v % glycerol, 200 mM NaCl, 20 mM HEPES pH 7.5) at −80°C. Library coverage was confirmed by 250 bp paired end Illumina deep sequencing, with statistics reported in Table S5.

S RBD escape mutants are identified by a competitive assay between a nAb and soluble ACE2 as fully described in Francino-Urdaniz et al. (2021). Briefly, yeast cells are grown in SDCAA for 4hat 30°C, pelleted, and then induced in SGCAA for 22hat 22°C. Cells are washed thoroughly in PBSA (PBS containing 1 g/L BSA) and then 3 × 10^7^ induced EBY100 yeast cells displaying S RBD were labelled with 10 μg/mL nAb IgG for 30 min at room temperature with mixing by pipetting every 10 min in PBSA. The same cells were labelled with 75 nM chemically biotinylated ACE2, in the same tube, for 30 min at room temperature in PBSA with mixing by pipetting every 10 min. The cells were centrifuged and washed with 1mL PBSA. Cells were then labeled with 1.2 μL FITC, 0.5 μL SAPE and 98.3 μL PBSA for 10 min at 4°C. Cells were centrifuged, washed with 1mL PBSF, resuspended to 1 mL PBSA and sorted using FACS. Two additional populations were sorted: a reference population containing only an FSC/SSC gate for isolation of yeast cells (see below) and a control population of library not competitively labelled.

To discriminate cell populations FACS gates are used as shown in Figure S7: an FSC/SSC gate for isolation of yeast cells, FSC-H/FSC-A gate to discriminate single cells, a FSC-A/FITC+ gate selects the cells displaying the RBD on their surface and the top 2% of cells by a PE^+^/FITC^+^ gate is collected. At least 2.0 × 10^5^ cells are collected and recovered in SDCAA with 50 μg/mL Kanamycin and 1× Pen/Strep for 30 h at 30°C. The collected DNA is sequenced using 250 bp PE on an Illumina MiSeq and analyzed with the code developed by Francino-Urdaniz et al. (2021). Outputs from the code are a per-mutation enrichment ratio defined as the log_2_-transform of the change in frequency of the selected population relative to the reference population and a false discovery rate (FDR) as previously described (Francino-Urdaniz et al., 2021).

#### Animal study

Golden Syrian hamsters were provided by Charles River Laboratories (CRL:LVG(SYR)) and housed at the Scripps Research Institute. Animals were infused with antibodies intraperitoneally as described previously (Rogers et al., 2020). The Scripps Research Institutional Animal Care and Use Committee (IACUC) approved all experimental procedures involving all the animals in accordance with Protocol #20-0003.

#### Viral load measurements - Plaque assay

SARS-CoV-2 titers were measured by homogenizing lung tissue in DMEM 2% FCS using 100 μm cell strainers (Myriad, 2825–8367). Homogenized organs were titrated 1:10 over 6 steps and layered overVero-E6 cells. After 1 h of incubation at 37°C, a 1% methylcellulose in DMEM overlay was added, and the cells were incubated for 3 days at 37°C. Cells were fixed with 4% PFA and plaques were counted by crystal violet staining.

### Quantification and statistical analysis

Statistical analysis was performed using Graph Pad Prism 8 for Mac, Graph Pad Software, San Diego, California, USA. Groups of data were compared using several methods including the grouped parametric One-Way ANOVA test and the grouped non-parametric Kruskall-Walli test. Data were considered statistically significant at p < 0.05.

## Data Availability

• CryoEM maps and models have been deposited to the Electron Microscopy Data Bank under accession codes EMD-24693, EMD-24694, EMD-24695, EMD-24696, EMD-24697 and EMD-24699, and the Protein Data Bank under accession codes 7ru1, 7ru2, 7ru3, 7ru4, 7ru5 and 7ru8. • Raw deep sequencing reads for escape mutant analysis have been deposited in the Sequencing Read Archive under accession numbers (SAMN20094129-SAMN20094136). • Sequences of engineered antibodies in this manuscript have been deposited in GenBank with accession numbers ON585716-ON585785. • This paper does not report original code. • Any additional information required to reanalyze the data reported in this paper is available from the lead contact upon request
