# Supplementary material for: Engineering SARS-CoV-2 neutralizing antibodies for increased potency and reduced viral escape pathways
Source: iScience. 2022 Aug 11;25(9):104914. doi: 10.1016/j.isci.2022.104914 (PMC9367177; doi:10.1016/j.isci.2022.104914)
Supplement: Document S1. Figures S1–S10 and Tables S1–S5 [file mmc1.pdf]

iScience, Volume *25*

Supplemental information

Engineering SARS-CoV-2 neutralizing antibodies for increased potency and reduced viral escape pathways

Fangzhu Zhao, Celina Keating, Gabriel Ozorowski, Namir Shaabani, Irene M. Francino-Urdaniz, Shawn Barman, Oliver Limbo, Alison Burns, Panpan Zhou, Michael J. Ricciardi, Jordan Woehl, Quoc Tran, Hannah L. Turner, Linghang Peng, Deli Huang, David Nemazee, Raiees Andrabi, Devin Sok, John R. Teijaro, Timothy A. Whitehead, Andrew B. Ward, Dennis R. Burton, and Joseph G. Jardine

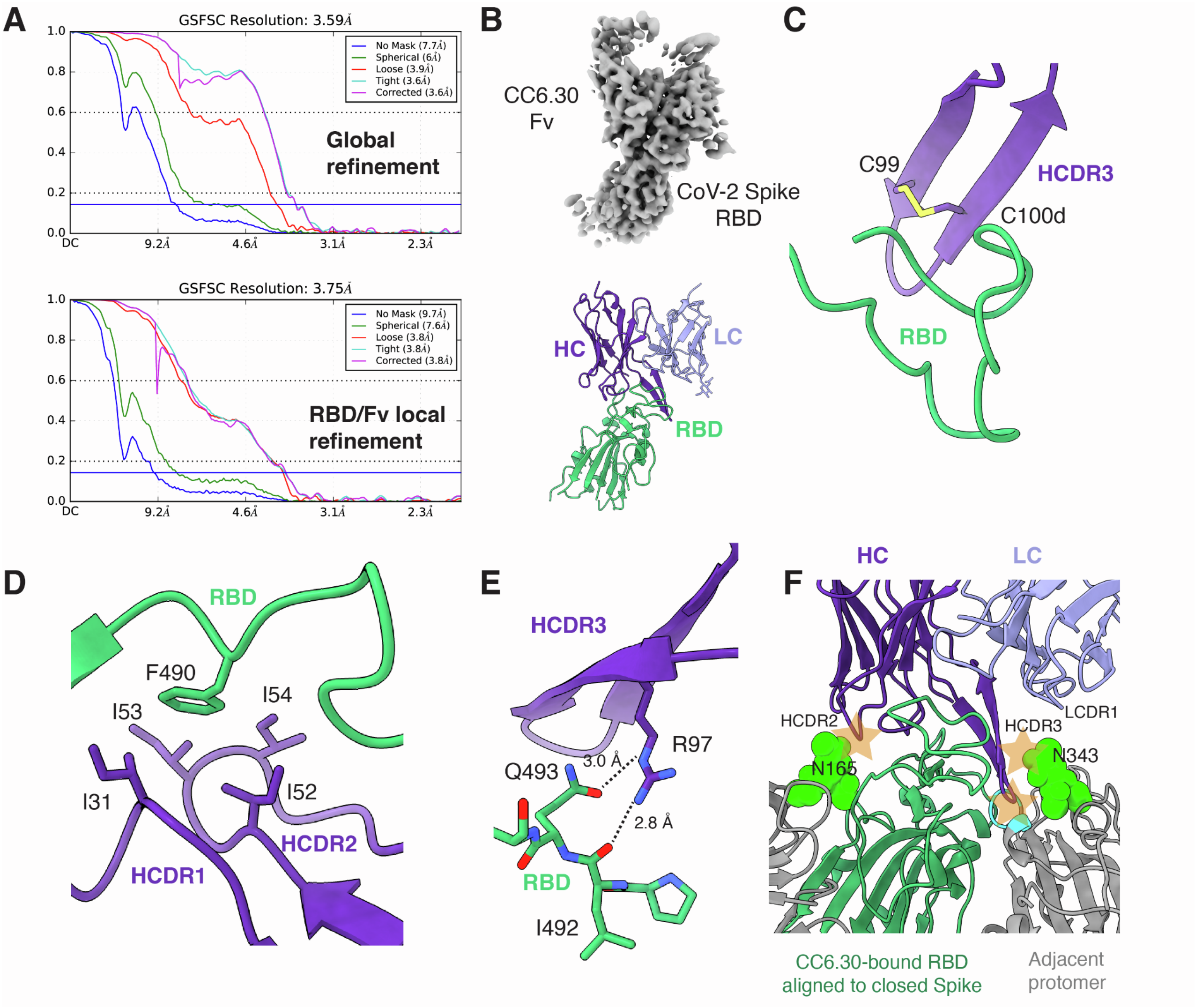

Figure S1. CryoEM reconstruction of nAb CC6.30 in complex with SARS-CoV-2 HPM7 Spike. Related to Figure 1. (**A**) Fourier shell correlation (FSC) of global refinement (*top*) and RBD/Fv local refinement (*bottom*) of the CC6.30:HPM7 complex. (**B**) Local refinement map (*top*) and model (*bottom*). (**C**) CC6.30 HCDR3 intrachain disulfide bond. (**D**) Hydrophobic interactions between RBD F490 and multiple CC6.30 heavy chain residues. (**E**) Putative hydrogen bonds (dashed lines) between CC6.30 HCDR3 R97 and RBD. (**F**) Superposition of RBD:CC6.30 onto a closed (3 down RBDs) Spike predicts multiple clashes (orange stars) with glycans (N165, N343) and peptide (residues 367-371; cyan) with the adjacent protomer.

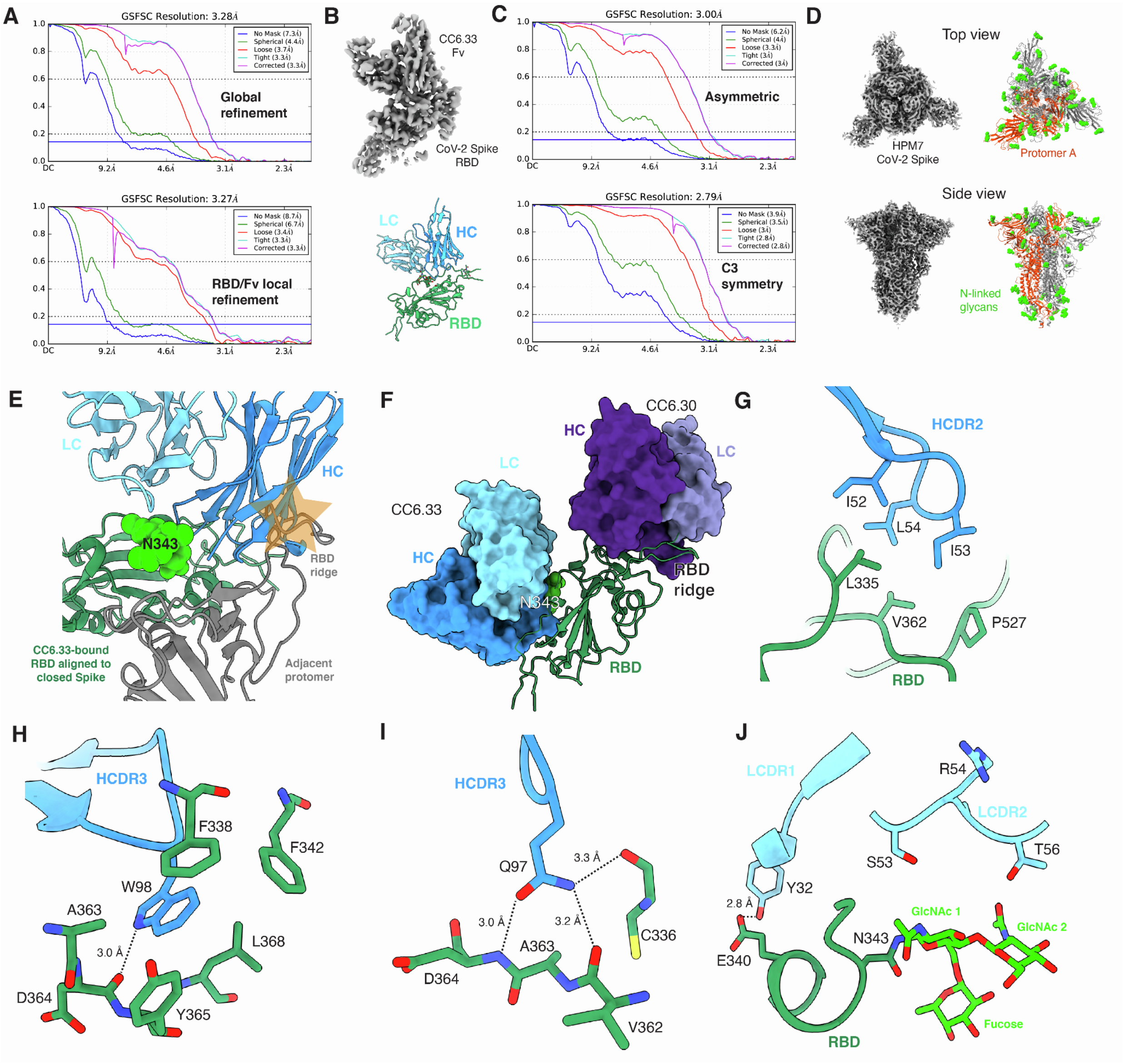

Figure S2. CryoEM reconstruction of nAb CC6.33 in complex with SARS-CoV-2 HPM7 Spike. Related to Figure 2. (**A**) Fourier shell correlation (FSC) of global refinement (*top*) and RBD/Fv local refinement (*bottom*) of the CC6.33:HPM7 complex. (**B**) Local refinement map (*top*) and model (*bottom*). (**C**) Fourier shell correlation (FSC) of asymmetric (*top*) and C3-symmetric (*bottom*) ligand-free HPM7 SARS-CoV-2 Spike reconstructions. (**D**) Map and model of ligand-free HPM7 SARS-CoV-2 Spike C3-symmetric reconstruction. (**E**) Superposition of RBD:CC6.33 onto a closed (3 down RBDs) Spike predicts a clash with the RBD ridge of an adjacent protomer that is relieved with slight opening of the apex. (**F**) Modeling of CC6.30 and CC6.33 on the same RBD reveals non-

overlapping epitopes. (**G**) Hydrophobic packing between CC6.33 HCDR2 and the RBD. (**H**) Interactions between CC6.33 HCDR3 Trp98 and the RBD. (**I**) Putative hydrogen bonding between CC6.33 HCDR3 Gln97 and the RBD. (**J**) Interface between CC6.33 LCDR1 and LCDR2, and RBD, including glycan N343.

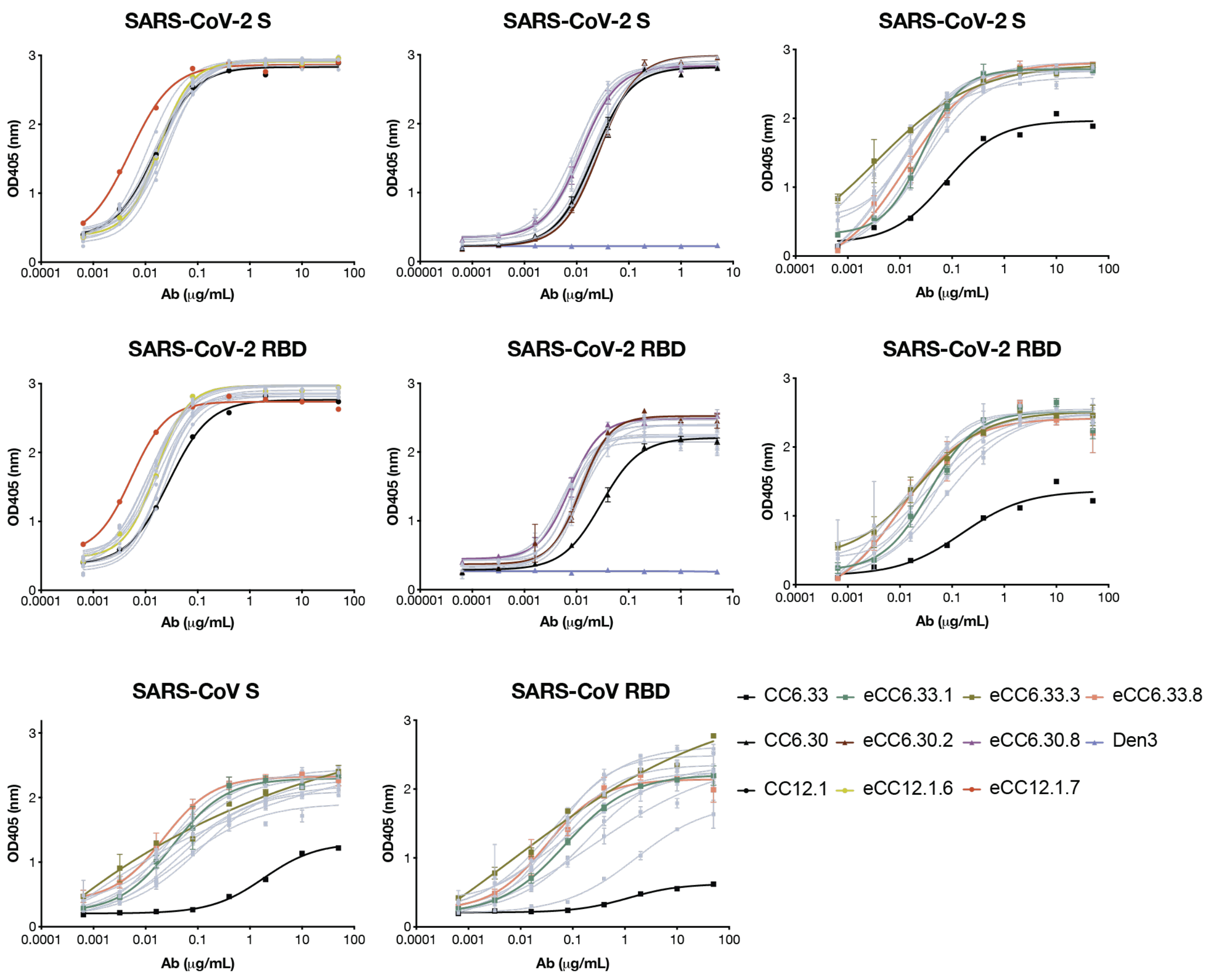

Figure S3. Engineered antibody variants ELISA binding to SARS-CoV-2 and SARS-CoV-1 S/RBD proteins. Related to Figure 3. Parental and engineered Abs were evaluated binding against his-tagged recombinant S/RBD protein. Parental mAbs were colored in black while some key enAbs were colored according to the key. The rest of enAbs were colored in grey. Data are represented as mean ± SD.

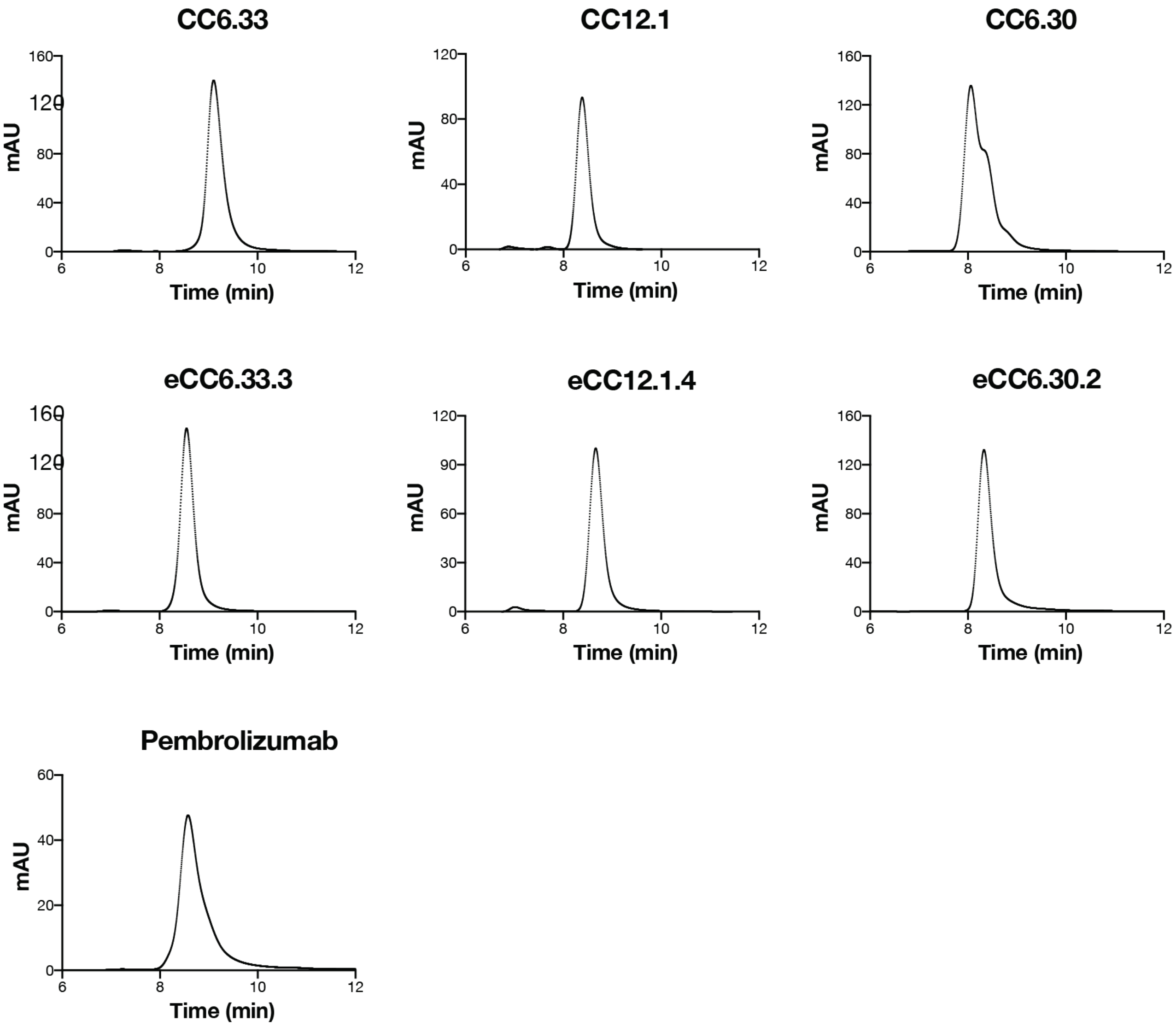

Figure S4. Size exclusion chromatography analysis of parental and engineered SARS-CoV-2 mAbs. Related to Figure 3. The antibodies were analyzed by size exclusion chromatography using the 1260 Infinity II (Agilent). 15 uL of each antibody was injected into the TSKgel SuperSW mAb column at 2 mg/mL. An FDA-approved mAb Pembrolizumab was included as control.

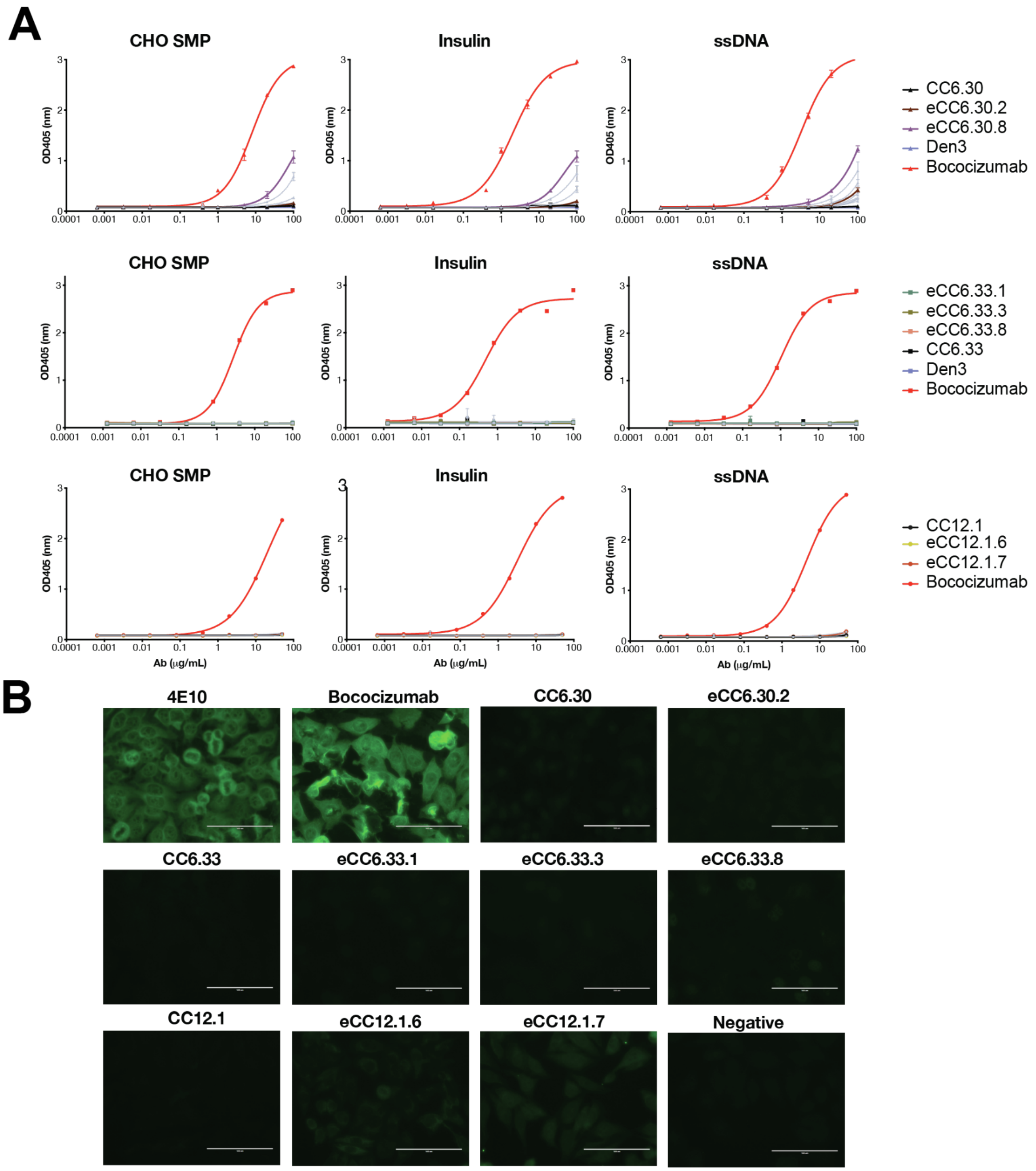

Figure S5. Polyreactivity of parental and enhanced nAbs by SPR. Related to Figure 3. (**A**) ELISA of eCC6.30, eCC6.33, eCC12.1 variants and parental clones to CHO solubilized membrane proteins, human insulin, and ssDNA. Bococizumab serves as positive control while Den3 serves as negative control. Data are represented as mean and standard deviation. (**B**) HEp2 epithelial cells staining with mAbs at 100 ug/mL. 4E10 and Bococizumab serve as positive control.

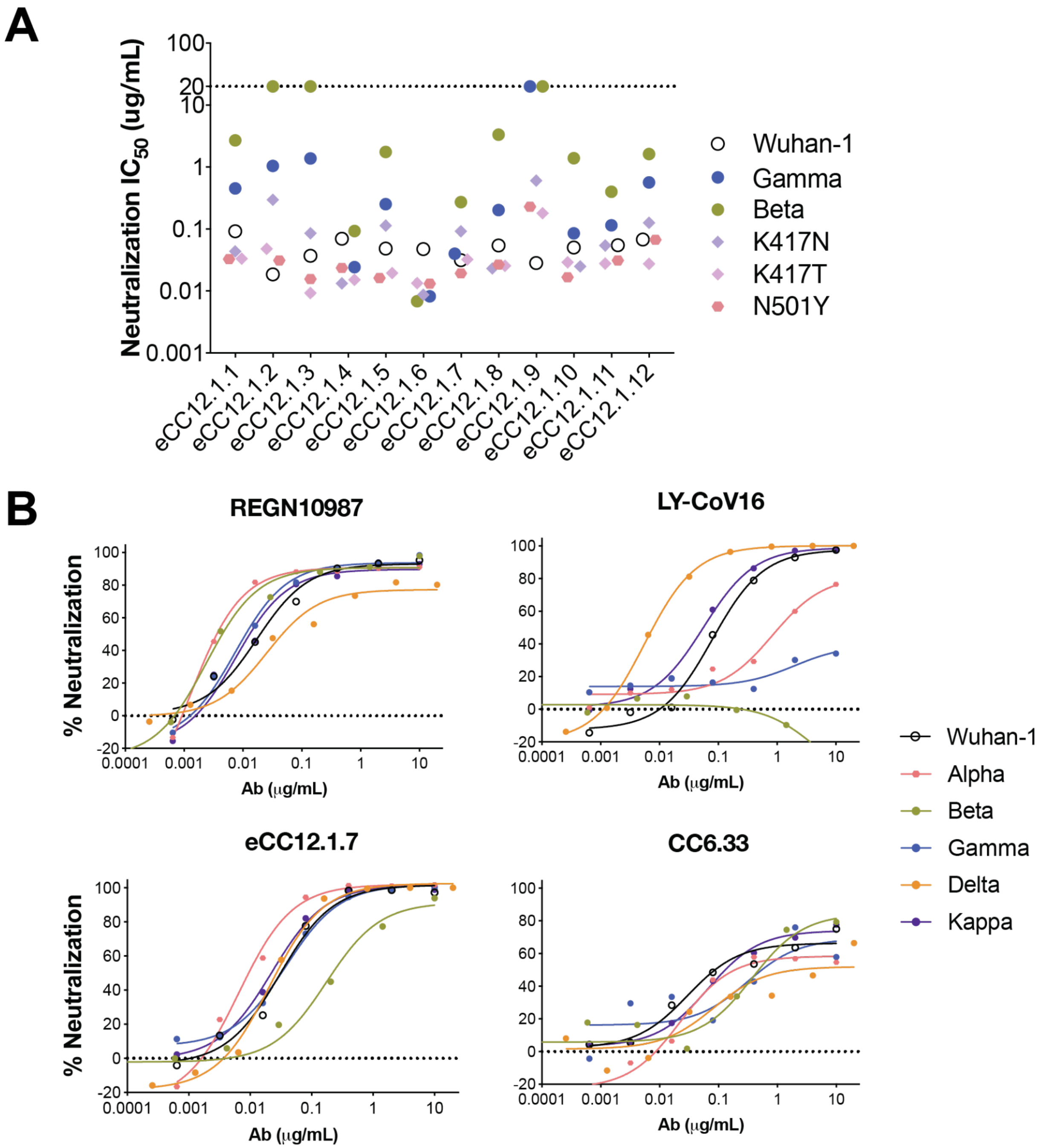

Figure S6. Neutralization of mAbs against SARS-CoV-2 Variants. Related to Figure 4. (**A**) Neutralization IC_{50} of 12 eCC12.1 variants against Wuhan-1, K417N, K417T, N501Y, Beta, and Gamma VOIs. Antibody started at 20 ug/mL and was tested in duplicates. (**B**) Neutralization curves of REGN10987, LY-CoV16, CC6.33 and eCC12.1.7 against Wuhan-1 as well as VOCs. Virus strains were colored according to the key.

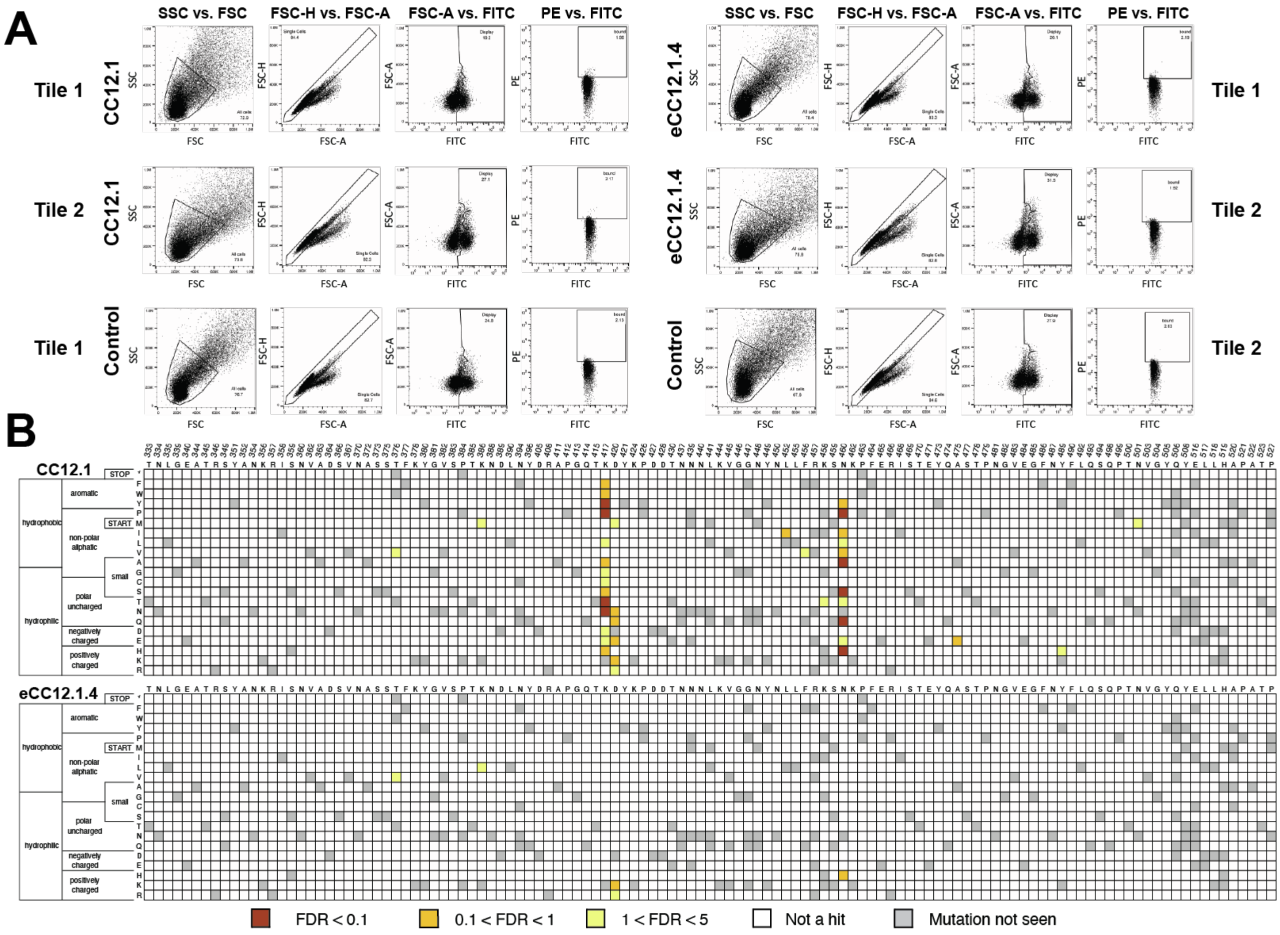

Figure S7. Identification of SARS-CoV-2 RBD escape mutants using yeast screening. Related to Figure 5. (**A**) Gating strategy and gates set for the escape mutant analysis for CC12.1 and eCC12.1.4. Control is the population of yeast cells without labeling by either biotinylated ACE2 or a given nAb. (**B**) Heatmap showing predicted RBD (residues 333-527) escape mutants for CC12.1 and eCC12.1.4 in yellow to burnt orange with varying levels of confidence according to the key.

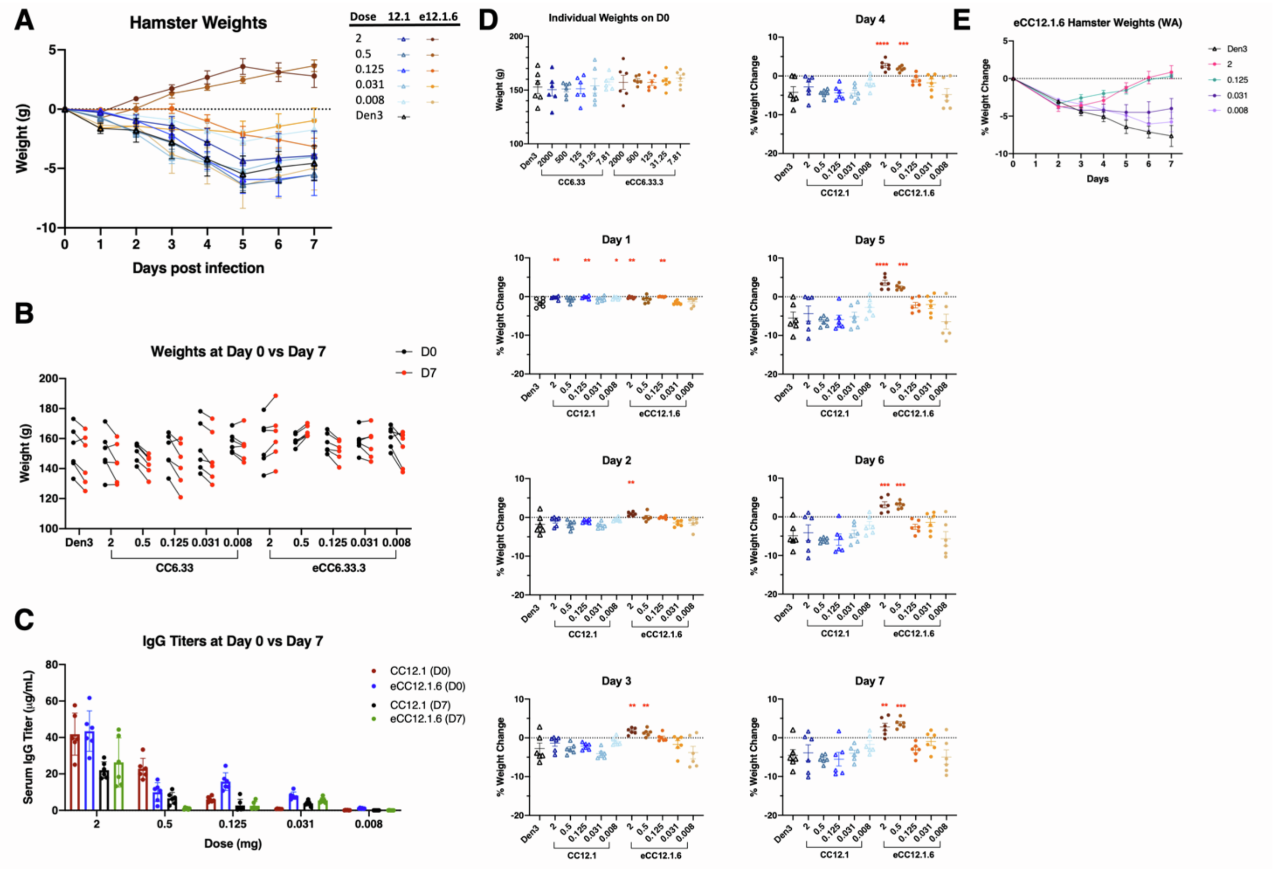

Figure S8. Supplemental Animal Protection Studies, CC12.1 and eCC12.1.6. **Related to Figure 6.** (**A**) Weight trends of all groups included in the CC12.1 vs eCC12.1.6 prophylactic protection study. (**B**) Weights of animals at time of challenge (Day 0) compared to weights at time of sacrifice (Day 7). (**C**) Serum human IgG concentration at time of infection (Day 0) compared to sacrifice (Day 7). (**D**) Percent weight loss by day compared to weights recorded at time of infection at day 0. (**E**) Weight trend of animals administered with eCC12.1.6 and subsequently challenged with 1×10^5 PFU of SARS-CoV-2 (USA-WA1/2020) three days later. Data are represented as mean ± SD.

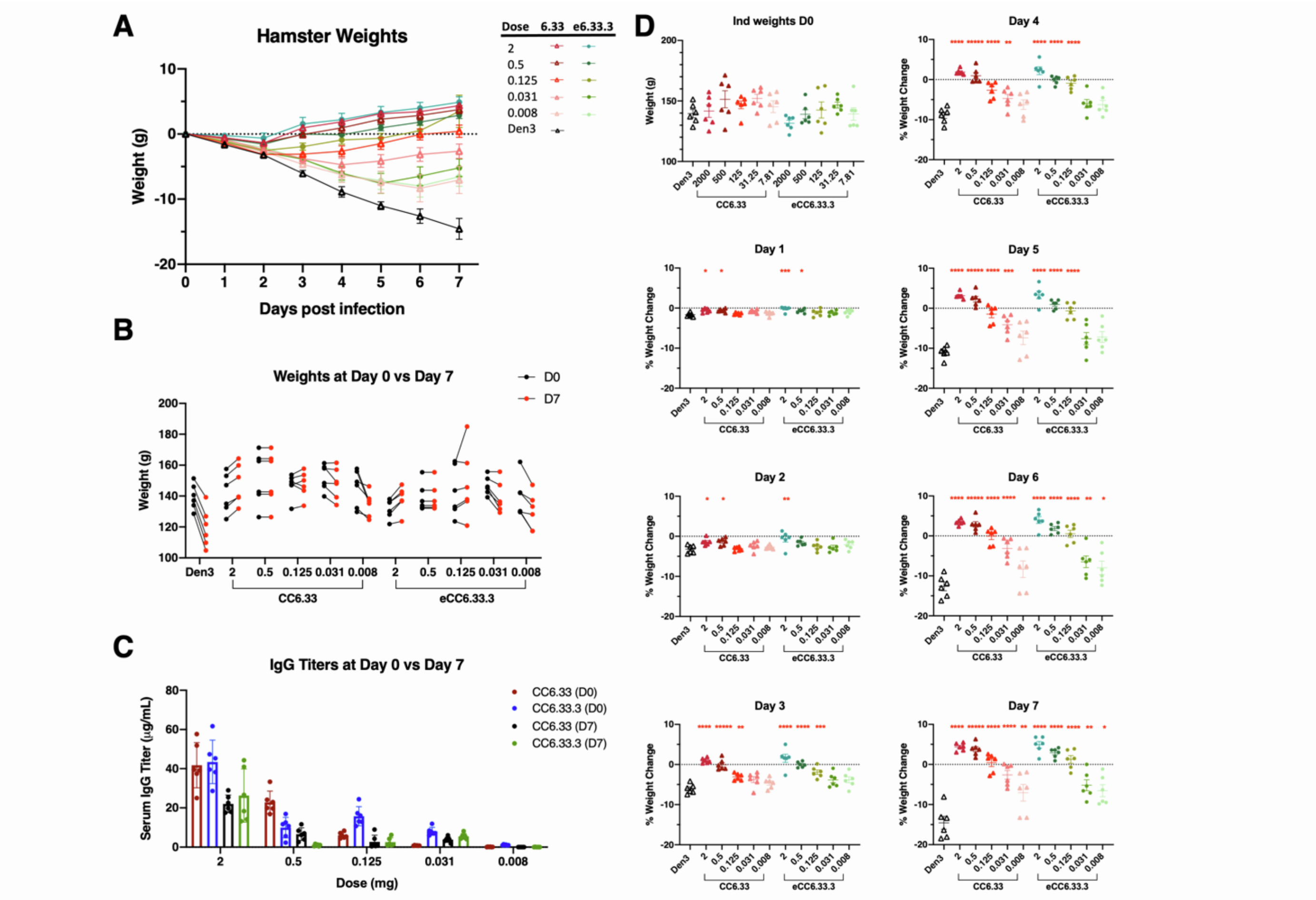

Figure S9. Supplemental Animal Protection Studies, CC6.33 and eCC6.33.3. Related to Figure 6. (**A**) Weight trends of all groups included in the CC6.33 vs eCC6.33.3 protection study. (**B**) Weights of animals at time of challenge (Day 0) compared to weights at time of sacrifice (Day 7). (**C**) Serum human IgG concentration at time of infection (Day 0) compared to sacrifice (Day 7). (**D**) Percent weight loss by day compared to individual weights recorded at time of infection at day 0. Data are represented as mean ± SD.

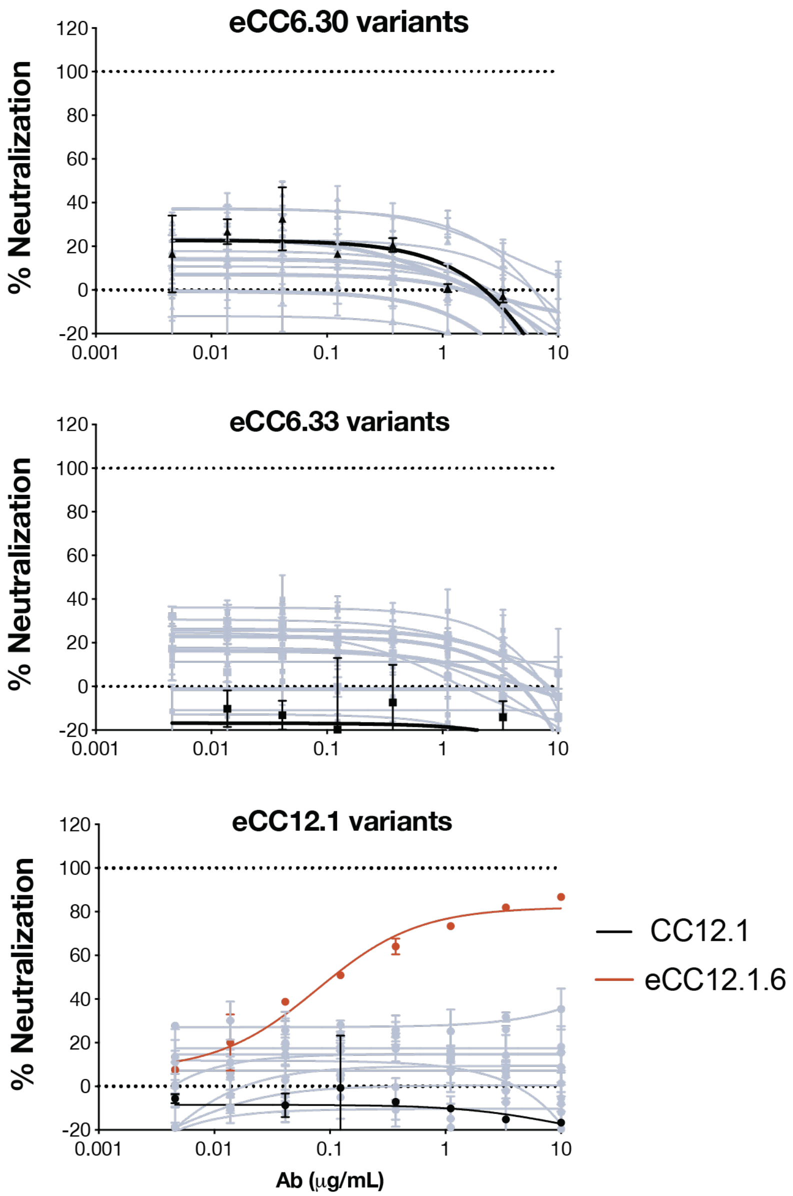

Figure S10. Neutralization of enAbs against the Omicron variant. Related to Figure 10. Neutralization curves of eCC6.33, eCC6.30, and eCC12.1 variants against pseudotyped Omicron VOC. Parental antibodies were highlighted in black whereas enAbs were in grey. eCC12.1.6 was highlighted according to the key. Data are represented as mean ± SD.

Table S1. CryoEM data collection, refinement and model building statistics (CC6.30 dataset). Related to Figure 1.

**Map**	**CC6.30 Fab + SARS-CoV-2 S HPM7 (Non-uniform refinement)**	**CC6.30 Fab + SARS-CoV-2 S HPM7 (RBD/Fv local refinement)**
EMDB	EMD-24697	EMD-24699
**Data collection**		
Microscope	FEI Titan Krios	FEI Titan Krios
Voltage (kV)	300	300
Detector	Gatan K2 Summit	Gatan K2 Summit
Recording mode	Counting	Counting
Nominal magnification	29,000	29,000
Movie micrograph pixel size (Å)	1.03	1.03
Dose rate (e^-/[(camera pixel)*s])	5.414	5.414
Number of frames per movie micrograph	39	39
Frame exposure time (ms)	250	250
Movie micrograph exposure time (s)	9.75	9.75
Total dose (e^-/Å^2)	50	50
Defocus range (µm)	-0.6 to -1.5	-0.6 to -1.5
**EM data processing**		
Number of movie micrographs	3,348	3,348
Number of molecular projection images in map	55,248	94,532
Symmetry	C1	C1
Map resolution (FSC 0.143; Å)	3.6	3.8
Map sharpening B-factor (Å^2)	-79.9	-50
**Structure Building and Validation**		
Number of atoms in deposited model		
SARS-CoV-2 S protein	24,577	1,388
Glycans	560	42
CC6.30 Fv	3,536	1,768
MolProbity score	0.91	1.19
Clashscore	0.81	0.96
Map correlation coefficient	0.72	0.72
EMRinger score	2.57	2.53
RMSD from ideal		
Bond length (Å)	0.02	0.02
Bond angles (˚)	1.81	1.88
Ramachandran plot		
Favored (%)	97.12	93.92
Allowed (%)	2.88	6.08
Outliers (%)	0	0
Side chain rotamer outliers (%)	0.06	0
PDB	7ru5	7ru8

Table S2. CryoEM data collection, refinement and model building statistics (CC6.33 dataset). Related to Figure 2.

**Map**	**CC6.33 IgG + SARS-CoV-2 S HPM7 (Non-uniform refinement)**	**CC6.33 IgG + SARS-CoV-2 S HPM7 (RBD/Fv local refinement)**	**SARS-CoV-2 S HPM7 (C3 symmetry)**	**SARS-CoV-2 S HPM7 (C1 symmetry)**
EMDB	EMD-24695	EMD-24696	EMD-24693	EMD-24694
**Data collection**				
Microscope	FEI Titan Krios	FEI Titan Krios	FEI Titan Krios	FEI Titan Krios
Voltage (kV)	300	300	300	300
Detector	Gatan K2 Summit	Gatan K2 Summit	Gatan K2 Summit	Gatan K2 Summit
Recording mode	Counting	Counting	Counting	Counting
Nominal magnification	29,000	29,000	29,000	29,000
Movie micrograph pixel size (Å)	1.03	1.03	1.03	1.03
Dose rate (e^-/[(camera pixel)*s])	5.414	5.414	5.414	5.414
Number of frames per movie micrograph	39	39	39	39
Frame exposure time (ms)	250	250	250	250
Movie micrograph exposure time (s)	9.75	9.75	9.75	9.75
Total dose (e^-/Å^2)	50	50	50	50
Defocus range (μm)	-0.5 to -1.5	-0.5 to -1.5	-0.5 to -1.5	-0.5 to -1.5
**EM data processing**				
Number of movie micrographs	3,465	3,465	3,465	3,465
Number of molecular projection images in map	47,461	96,012	55,575	55,575
Symmetry	C1	C1	C3	C1
Map resolution (FSC 0.143; Å)	3.3	3.3	2.8	3
Map sharpening B-factor (Å^2)	-59.4	-33.9	-66.9	-54.6
**Structure Building and Validation**				
Number of atoms in deposited model				
SARS-CoV-2 S protein	24,175	1,610	25,737	25,778
Glycans	584	52	756	868
CC6.33 Fv	3,398	1,720	0	0
MolProbity score	1	0.89	0.91	0.83
Clashscore	1.04	0.6	0.77	0.46
Map correlation coefficient	0.81	0.8	0.83	0.83
EMRinger score	2.55	3.98	5.05	4.15
RMSD from ideal				
Bond length (Å)	0.02	0.02	0.02	0.02
Bond angles (˚)	1.85	1.9	1.77	1.78
Ramachandran plot				
Favored (%)	96.82	96.9	96.97	97.04
Allowed (%)	3.18	3.1	3.03	2.96
Outliers (%)	0	0	0	0
Side chain rotamer outliers (%)	0.32	0.55	0	0.14
PDB	7ru3	7ru4	7ru1	7ru2

Table S3. CDR loop sequences, RBD binding affinity, and neutralization potency of parent and engineered antibodies. Related to Figure 3.

mAb ID	HCDR Sequence			LCDR Sequence			SPR against SARS-CoV-2 RBD			PSV Neutralization Potency (ug/mL)		
	H-CDR1	H-CDR2	H-CDR3	L-CDR1	L-CDR2	L-CDR3	ka [1/Ms]	kd [1/s]	KD [M]	SARS1	SARS2	MPN
**CC12.1**	SGLTVSSNYMS	VIYSGGST	ARDLDVYGLDV	ASQGISSY	YAASTLQS	QQLNSYPPKF	6.95E+05	4.12E-03	5.92E-09	>10	0.017	100%
eCC12.1.1	SGLTVSSNYMV	VIYSGGSI	ARDLDVYGLDV	ASQGISSY	YAASTLQW	QQLNSYPPPF	7.81E+05	7.76E-05	9.92E-11	>10	0.012	100%
eCC12.1.2	FGLTVSSNYMS	QIYSGGST	ARDLDVYGVDV	ASQGISSY	YAASTIQS	QQLNSYPPPF	7.51E+05	2.35E-04	3.13E-10	>10	0.014	100%
eCC12.1.3	SGLTVSSNYMN	VIYSGGST	ARDLDVYGVDV	ASQGISHY	YAASYLQS	QQLNSYPPKP	7.08E+05	1.11E-04	1.57E-10	>10	0.009	100%
eCC12.1.4	SGLTVSLNYMS	EIYSGGST	ARDLDVYGLDV	ASQGISSY	YAASTLQW	QQLNSYPPPF	8.21E+05	2.35E-04	2.86E-10	>10	0.013	100%
eCC12.1.5	SGLTVSSNYMV	VIYPGGST	ARDLDVYGIDV	ASQGISNY	YAASTLQW	VQLNSYPPKF	3.93E+06	1.29E-04	3.28E-11	>10	0.007	100%
eCC12.1.6	SGLTVSSNYMA	VIYAGGST	ARDLDVYGIDV	ASQGISWY	YAASTLQW	QQLNSYPPKS	1.04E+06	9.62E-05	9.26E-11	>10	0.008	100%
eCC12.1.7	SGLTVSSNYMT	VIYAGGST	ARDLDVYGVDV	ASQGISRY	YAASTLQY	QQLNSYPPKY	8.48E+05	1.65E-04	1.95E-10	>10	0.005	100%
eCC12.1.8	SGLTVSSNYML	LIYSGGST	ARDLDVYGVDV	ASQGISSY	YAASTLQR	QQLNSYPPPF	7.49E+05	9.36E-05	1.25E-10	>10	0.011	100%
eCC12.1.9	SGLWVSSNYMS	QIYSGGST	ARDLDVYGIDV	ASQGISHY	YAASNLQS	QQLNSYPPPF	6.06E+05	1.99E-04	3.28E-10	>10	0.020	100%
eCC12.1.10	FGLTVSSNYMS	QIYSGGST	ARDLDVYGVDV	ASQGISSY	YAASTIQS	QQLNSYPPPF	2.73E+06	6.04E-05	2.22E-11	>10	0.009	100%
eCC12.1.11	SGLTVSSNYMV	QIYSGGST	ARDLDVYGLDV	ASQGISSY	YAASTLQH	QQLNSYPPPF	9.04E+05	1.01E-04	1.11E-10	>10	0.008	100%
eCC12.1.12	SGLTVSSNYMT	VIYPGGST	ARDLDVFGLDV	VSQGISSY	YFASTLQS	QQLNSYPPPF	3.64E+07	9.46E-05	2.60E-12	>10	0.017	100%
**CC6.33**	KASGGTFSSSAIS	GIIPILDITN	LRNQWDLLV	RASQSVSSSYLA	YGASSRAT	QHYGSSLWT	5.93E+04	1.52E-02	2.57E-07	2.269	0.228	81%
eCC6.33.1	KASGGIFSSSAIS	GIIPILDISN	LTNQWDLLV	RASQRVSSSYLA	YGAESRAT	QHYGSSLWT	5.18E+04	7.91E-04	1.53E-08	0.024	0.006	100%
eCC6.33.2	KAPGGTFSSSAIS	TIIPILDITN	QRNQWDLLV	RASQSVSASYLA	YGAESRAT	QHYGSSRWT	8.97E+04	1.12E-03	1.25E-08	0.031	0.008	98%
eCC6.33.3	KAPGGTFSSSAIS	GIIPILDITH	LRNQWDLLV	RASQDVSSSYLA	YGAESRAT	QHYGSSEWT	1.16E+05	9.53E-04	8.18E-09	0.023	0.008	95%
eCC6.33.4	KASGGIFSSSAIS	GIIPILDITN	LTNQWDLLV	RASQSVSSSYLA	YGAESRAT	QHYGSSAWT	-	-	-	0.037	0.016	95%
eCC6.33.5	KAVGGTFSSSAIY	GIIPILDIAN	LRNQWDLVV	HASQSVSSSYLA	YGAESRAT	QHYGSSTWT	2.37E+05	4.11E-03	1.73E-08	0.016	0.004	95%
eCC6.33.6	KAPGGTFSSSAIS	GIIPILDITT	LRNQWDLLI	RASQSVSSHYLA	YGASSRYT	QHYGSSHWT	1.26E+05	1.67E-03	1.33E-08	0.012	0.020	98%
eCC6.33.7	KASGGIFSSSAIS	GIIPILDYTN	LRNQWDLLV	RASQEVSSSYLA	YGAESRAT	QHYGSSAWT	2.20E+05	1.67E-03	7.60E-09	0.094	0.017	100%
eCC6.33.8	KVSGGTFSSSAIS	KIIPILDITN	LRNQWDLLV	RASQSVSSHYLA	YGAESRAT	QHYGSSPWT	1.74E+05	4.25E-04	2.44E-09	0.013	0.010	100%
eCC6.33.9	KSSGGTFSSSAYS	TIIPILDITN	QRNQWDLIV	RASQWVSSSYIA	YGAESRAT	QHYGSSSWT	1.53E+05	2.18E-03	1.43E-08	0.054	0.014	97%
eCC6.33.10	KAPGGTFSSSAIS	GIIPILDITH	LRNQWDLLV	RASQSVSSSYLA	YGAESRAT	QHYGSSVWT	1.84E+05	1.70E-03	9.24E-09	0.010	0.012	99%
eCC6.33.11	KISGGYFSSSAIS	GIIPVLDITN	LTNQWDLLV	SASQSVSSSYLA	YGAESRAT	QHYGSSHWT	1.58E+05	3.76E-04	2.38E-09	0.020	0.010	100%
eCC6.33.12	KASGGLFSSSAIS	GIIPILDITN	LTNQWDLLV	RASQSVSSSYLA	YGAESRAT	QHYGSSRWT	1.70E+05	1.38E-03	8.11E-09	0.061	0.013	99%
**CC6.30**	KASGGTFSIYAIT	GIIPIIGTAN	RDFRYCSSTRCYFWF	RASQSISSYL	YAASSLQS	QQSYSTPRT	2.52E+06	4.30E-03	1.71E-09	>10	0.006	100%
eCC6.30.1	KASGGTFSIYAIT	NIIPIIGTAN	RDFRYCSSTRCYFWF	RASQSIQSYL	YTASSLQS	QQSYSTPRS	1.27E+06	7.19E-05	5.66E-11	>10	0.014	100%
eCC6.30.2	KASGGAFSIYSIT	GIIPIRGTAN	RDFRYCSSDRCYFWF	RASQSIGSYL	YTASSLQS	QQSYSTPRT	1.46E+06	5.93E-05	4.06E-11	>10	0.016	100%
eCC6.30.3	KVSGGWFSIYAIT	GIIPIQGTAN	LDFRYCSSTRCYFWF	RASQSISEYL	YTASSLQS	QQSYSTPRT	1.54E+06	1.15E-04	7.47E-11	>10	0.041	100%
eCC6.30.4	KASGGTFDIYAIT	GIIPIRGTAN	RDFRYCSSTRCYFWF	RASQSISEYL	YRASSLQS	QQSYSTPRT	1.12E+06	2.78E-04	2.49E-10	>10	0.013	100%
eCC6.30.5	KASGGAFSIYAIT	GIIPIIGTAN	RDFRYCESTRCYFWF	HASQSISSYL	YTASSLQS	QQSYSTPRT	1.02E+06	1.13E-04	1.10E-10	>10	0.026	100%
eCC6.30.6	KASGGTFSIYALT	GIIPIQGTAN	RDFRYCSGTRCYFWF	RASQSISTYL	YSASSLQS	QQSYSTPRT	1.09E+06	3.98E-04	3.64E-10	>10	0.013	100%
eCC6.30.7	KASGGTFSIYAIT	NIIPIIGTAN	YDFRYCSSTRCYFWF	RASQSISEYL	YQASSLQS	GQSYSTPRT	1.40E+06	2.19E-04	1.56E-10	>10	0.015	100%
eCC6.30.8	KASGGPFSIYAIT	GIIPIRGTAN	RDFRYCSSTRCYFWF	QASQSISSYL	YTASSLQS	QQSYSTPRT	1.16E+06	1.11E-04	9.57E-11	>10	0.010	100%
eCC6.30.9	KASGGIFSIYAIT	AIIPIIGTAN	RDFRYCSSTRCYFNF	RASQSISNYL	YTASSLQS	QQSYSTPRT	2.57E+06	2.02E-04	7.88E-11	>10	0.016	100%
eCC6.30.10	KASGGTFAIYAIT	NIIPIIGTAN	RDFRYCSSTQCYFWF	RASQPISSYL	YTASSLQS	QQSYSTPRT	1.47E+06	4.29E-05	2.91E-11	>10	0.017	100%
eCC6.30.11	KASGGAFSIYAIT	GIIPIRGTAN	RDFRYCSETRCYFWF	RASQDISSYL	YRASSLQS	QQSYSTPRS	2.71E+06	1.27E-04	4.71E-11	>10	0.025	100%

Summary table of parental CC12.1, CC6.33, CC6.30 and 35 engineered antibodies with sequences of 6 CDR loops, binding affinity against SARS-CoV-2 RBD, SARS-CoV-1/2 neutralization potency and maximum percentage of neutralization (MPN). Mutations of engineered antibodies at CDR loops were highlighted in red. Antibodies were captured via Fc-capture to an anti-human IgG Fc antibody and varying concentrations of SARS-CoV-2 RBD were injected using a multi-cycle method. Association and dissociation rate constants calculated through a 1:1 Langmuir binding model using the BIAevaluation software. Neutralization assay was performed using pseudotyped SARS-CoV-1 and SARS-CoV-2 with Hela-hACE2 cell line. All antibodies were tested in duplicates.

Table S4. Surface plasmon resonance of SARS-CoV-2 nAbs against RBD variants. Related to Figure 4.

SPR against RBD		CC6.30	eCC6.30.2	REGN10933	CC12.1	eCC12.1.4	eCC12.1.7	LY-CoV16	CC6.33	eCC6.33.8	REGN10987
**Wuhan-1**	ka [1/Ms]	2.52E+06	1.46E+06	3.48E+06	6.96E+05	8.21E+05	8.48E+05	1.30E+06	5.93E+04	1.74E+05	1.76E+06
	kd [1/s]	4.30E-03	5.93E-05	4.31E-03	4.12E-03	2.35E-04	1.65E-04	1.87E-02	1.52E-02	4.25E-04	1.86E-02
	KD [M]	1.71E-09	4.06E-11	1.24E-09	5.92E-09	2.86E-10	1.95E-10	1.51E-08	2.57E-07	2.44E-09	1.06E-08
**K417N**	ka [1/Ms]	2.67E+06	1.01E+06	2.47E+06	-	5.44E+05	6.07E+05	-	2.20E+05	2.00E+05	3.73E+06
	kd [1/s]	3.86E-03	6.68E-04	3.89E-02	-	3.54E-03	3.01E-03	-	1.12E-02	3.20E-04	3.76E-02
	KD [M]	1.45E-09	6.60E-10	1.58E-08	-	6.51E-09	4.97E-09	-	5.07E-08	1.60E-09	1.08E-08
**K417T**	ka [1/Ms]	3.19E+06	8.67E+05	3.15E+06	-	5.11E+05	4.77E+05	-	1.51E+05	2.49E+05	3.37E+06
	kd [1/s]	2.03E-03	5.35E-04	4.85E-02	-	9.31E-04	6.65E-04	-	7.94E-03	6.10E-04	4.89E-02
	KD [M]	6.36E-10	6.18E-10	1.54E-08	-	1.82E-09	1.40E-09	-	5.26E-08	2.45E-09	1.45E-08
**L452R**	ka [1/Ms]	-	2.11E+06	3.96E+06	4.80E+05	7.34E+05	8.78E+05	6.75E+05	2.01E+05	2.24E+05	1.56E+06
	kd [1/s]	-	7.74E-03	7.74E-03	2.40E-03	8.07E-04	4.96E-04	1.09E-02	6.33E-03	2.64E-04	1.71E-02
	KD [M]	-	3.68E-09	1.38E-09	5.01E-09	1.10E-09	5.66E-10	1.61E-08	3.10E-08	1.18E-09	1.10E-08
**E484K**	ka [1/Ms]	-	-	2.03E+06	2.60E+06	8.41E+05	1.04E+06	4.41E+05	1.98E+05	1.80E+05	1.68E+06
	kd [1/s]	-	-	4.07E-02	4.52E-03	2.02E-04	1.20E-04	1.20E-02	8.75E-03	2.82E-04	1.79E-02
	KD [M]	-	-	2.00E-08	1.74E-09	2.41E-10	1.15E-10	2.72E-08	4.43E-08	1.57E-09	1.06E-08
**E484Q**	ka [1/Ms]	-	1.82E+06	2.46E+06	4.02E+05	9.23E+05	1.07E+06	9.50E+05	1.83E+05	1.87E+05	1.17E+06
	kd [1/s]	-	4.51E-03	1.70E-02	1.38E-03	5.37E-04	3.94E-04	1.56E-02	8.90E-03	2.55E-04	1.87E-03
	KD [M]	-	2.48E-09	7.00E-09	3.43E-09	5.82E-10	3.67E-10	1.64E-08	4.87E-08	1.36E-09	1.61E-09
**Alpha**	ka [1/Ms]	2.62E+06	9.82E+05	2.61E+06	3.58E+05	8.08E+05	8.86E+05	8.22E+05	2.20E+05	1.96E+05	1.66E+06
	kd [1/s]	6.91E-03	6.17E-04	3.36E-03	5.16E-03	4.43E-04	2.37E-03	7.01E-02	7.45E-03	2.67E-04	1.10E-02
	KD [M]	2.64E-09	6.28E-10	1.29E-09	1.44E-08	5.48E-10	2.67E-09	8.53E-08	3.39E-08	1.36E-09	6.63E-09
**Beta**	ka [1/Ms]	-	-	-	-	1.61E+05	2.18E+05	-	1.26E+05	1.10E+05	7.99E+05
	kd [1/s]	-	-	-	-	8.70E-03	4.50E-02	-	1.14E-02	2.78E-04	1.25E-02
	KD [M]	-	-	-	-	5.42E-08	2.07E-07	-	9.05E-08	2.53E-09	1.56E-08
**Gamma**	ka [1/Ms]	-	-	1.17E+05	-	7.15E-05	1.02E+06	-	1.41E+05	1.96E+05	2.84E+06
	kd [1/s]	-	-	1.23E-03	-	9.00E-03	3.62E-02	-	1.07E-02	2.63E-04	1.52E-02
	KD [M]	-	-	1.05E-08	-	1.26E-08	3.56E-08	-	7.57E-08	1.35E-09	5.33E-09
**Kappa**	ka [1/Ms]	-	-	3.18E+06	3.80E+05	8.57E+05	1.19E+06	1.27E+06	1.18E+05	2.22E+05	2.49E+06
	kd [1/s]	-	-	3.17E-02	1.36E-03	6.49E-04	2.12E-04	1.83E-02	1.22E-02	2.69E-04	1.72E-02
	KD [M]	-	-	9.97E-09	3.59E-09	7.57E-10	1.79E-10	1.44E-08	1.03E-07	1.22E-09	6.92E-09

Antibodies were captured via Fc-capture to an anti-human IgG Fc antibody and varying concentrations of Wuhan-1 RBD or SARS-CoV-2 RBD variants were injected using a multi-cycle method. Association and dissociation rate constants calculated through a 1:1 Langmuir binding model using the BIAevaluation software. Antibodies that did not bind to RBD variants or their binding curves did not fit into the model were shown as no values in the table.

Table S5. S RBD Single saturation mutagenesis library coverage statistics. Related to Figure 5.

	S RBD N343Q (Wuhan-1)	
Tile Number	Tile 1	Tile 2
Positions	333-436	437-537
Number of Designed Mutations	1120	1260
*E. coli t*ransformants Obtained from Nicking Saturation Mutagenesis	5.0E+05	1.5E+06
Yeast transformants Obtained from Homologous Recombination	8.0E+05	1.0E+06
Library Coverage Per Tile	97% (1089/1120)	92% (1161/1260)
Overall Library Coverage	94.5% (2250/2380)	
